# Modeling disrupted synapse formation in wolfram syndrome using hESCs-derived neural cells and cerebral organoids identifies Riluzole as a therapeutic molecule

**DOI:** 10.1038/s41380-023-01987-3

**Published:** 2023-02-07

**Authors:** Fei Yuan, Yana Li, Rui Hu, Mengting Gong, Mengyao Chai, Xuefei Ma, Jiaxue Cha, Pan Guo, Kaijiang Yang, Mushan Li, Minglu Xu, Qing Ma, Qiang Su, Chuan Zhang, Zhejin Sheng, Heng Wu, Yuan Wang, Wen Yuan, Shan Bian, Li Shao, Ru Zhang, Kaicheng Li, Zhen Shao, Zhen-Ning Zhang, Weida Li

**Affiliations:** 1 Translational Medical Center for Stem Cell Therapy and Institute for Regenerative Medicine, Shanghai East Hospital, Frontier Science Center for Stem Cell Research, School of Life Sciences and Technology, Tongji University, Shanghai, 200092 China; 2 Tsingtao Advanced Research Institute, Tongji University, Qingdao, 266071 China; 3 CAS Key Laboratory of Computational Biology, Shanghai Institute of Nutrition and Health, Chinese Academy of Sciences, Shanghai, 200031 China; 4 University of Chinese Academy of Sciences, Beijing, 100049 China; 5 QuietD Biotechnology, Ltd., Shanghai, 201210 China; 6 Shanghai Key Laboratory of Signaling and Disease Research, School of Life Sciences and Technology, Tongji University, Shanghai, 200092 China; 7 Department of Statistics, The Pennsylvania State University, University Park, PA 16802 USA; 8 School of Medicine, Tongji University, Shanghai, 200092 China; 9 Department of Psychosomatic Medicine, Shanghai Tongji Hospital, Tongji University School of Medicine, Shanghai, 200092 China; 10 Department of Neurology and Department of Neurosurgery, State Key Laboratory of Biotherapy and Cancer Center, West China Hospital, Sichuan University and National Collaborative Innovation Center, Chengdu, Sichuan 610041 China; 11 Department of Stem Cell and Regenerative Biology, Harvard University, Cambridge, MA 02138 USA; 12 Department of VIP Clinic, Shanghai East Hospital, Tongji University School of Medicine, Shanghai, 200092 China; 13 Reg-Verse Therapeutics (Shanghai) Co. Ltd., Shanghai, 200120 China

**Keywords:** Neuroscience, Stem cells

## Abstract

Dysregulated neurite outgrowth and synapse formation underlie many psychiatric disorders, which are also manifested by wolfram syndrome (WS). Whether and how the causative gene *WFS1* deficiency affects synapse formation remain elusive. By mirroring human brain development with cerebral organoids, *WFS1*-deficient cerebral organoids not only recapitulate the neuronal loss in WS patients, but also exhibit significantly impaired synapse formation and function associated with reduced astrocytes. *WFS1* deficiency in neurons autonomously delays neuronal differentiation with altered expressions of genes associated with psychiatric disorders, and impairs neurite outgrowth and synapse formation with elevated cytosolic calcium. Intriguingly, *WFS1* deficiency in astrocytes decreases the expression of glutamate transporter *EAAT2* by NF-κB activation and induces excessive glutamate. When co-cultured with wildtype neurons, *WFS1*-deficient astrocytes lead to impaired neurite outgrowth and increased cytosolic calcium in neurons. Importantly, disrupted synapse formation and function in *WFS1*-deficient cerebral organoids and impaired neurite outgrowth affected by *WFS1*-deficient astrocytes are efficiently reversed with Riluzole treatment, by restoring *EAAT2* expression in astrocytes. Furthermore, Riluzole rescues the depressive-like behavior in the forced swimming test and the impaired recognition and spatial memory in the novel object test and water maze test in *Wfs1* conditional knockout mice. Altogether, our study provides novel insights into how *WFS1* deficiency affects synapse formation and function, and offers a strategy to treat this disease.

## Introduction

Wolfram syndrome (WS) is a recessive genetic disease manifested by juvenile-onset diabetes mellitus, optic nerve atrophy, hearing loss and wide spectrum of neurological disorders [1–5]. WS patients are also characterized by severe psychiatric manifestations, such as schizophrenia, anxiety, depression, psychosis, panic attack and mood wings [6–10]. *WFS1*, highly expressed in brain and pancreas, is the major causative gene of WS, and its deficiency presents in 90% WS patients [11, 12]. WS is caused by homozygous or compound heterozygous (both alleles are mutated, but the mutations are not identical) loss of function (LOF) variants in the *WFS1* gene, which are biallelic pathogenic variants and inherited in an autosomal recessive manner [12–14]. Patients with *WFS1* homozygous or compound heterozygous mutations suffer from typical clinical features of WS [15]. *WFS1* is of substantial importance for human mental health. However, due to ethical issues and scarcity of human samples, little is known of the underlying pathogenic mechanism.

During neurodevelopment to form neural connectivity, neurite outgrowth precedes and contributes to synapse formation by developing axon and dendrites [16–18]. Accumulative evidence suggests that dysregulated neurite outgrowth, synapse formation and synaptic function underlie psychiatric disorders, such as schizophrenia with reduced synapses and autism spectrum disorders with excess synapses [19–26]. Current studies on WS neuropathy mainly focus on investigating the role of *WFS1* in dysfunction and degeneration of neurons in *Drosophila*, fly, and mouse model [27–29]. However, the mechanisms of how *WFS1* deficiency impacts synapse formation underlying psychiatric disorders in WS remain elusive. Moreover, in *Drosophila* model of WS, knockdown of *wfs1* in both neurons and glial cells resulted in more severe behavioral deficits than knockdown of *wfs1* in neurons alone, indicating that interplay between neurons and astrocytes plays an essential role in WS neuropathogenesis [27]. Astrocytes constitute half of the cells in the brain, and have been considered as having an important pathogenic role in multiple neurodevelopmental and neurodegenerative disorders [30–33]. However, there is little evidence in whether and how *WFS1* deficiency in astrocytes impacts neurons in WS.

Considering the significant divergences in structure, cell types and cognitive capacity between brains of human and experimental animals, lack of proper human disease models limits understanding how *WFS1* deficiency contributes to psychiatric disorders in WS. Recently, various neural cells derived from isogenic human pluripotent stem cells are widely used to model human neurological or psychiatric disorders [23, 24], allowing investigation of pathogenesis in a fixed genetic background. Furthermore, technical breakthrough in generating human cerebral organoids from pluripotent stem cells holds promise to recapitulate human brain organogenesis and model neurodevelopmental disorders [34–38]. Taken together, these human pluripotent stem cell-based technological breakthroughs offer unprecedented opportunities to investigate whether and how *WFS1* deficiency affects synapse formation in human disease models with various neural cell types.

Here, we apply a multi-dimensional strategy of combining 3D cerebral organoids and 2D neural differentiation derived from human embryonic stem cells (hESCs) harboring *WFS1* deficiency. Our results reveal that *WFS1* deficiency significantly impairs synapse formation and function in cerebral organoids associated with decreased astrocytes. *WFS1* deficiency autonomously delays neuronal differentiation and affects synapse formation. Moreover, *WFS1* deficiency decreases the expression of glutamate transporter *EAAT2* by NF-κB activation and compromises glutamate clearance capacity of astrocytes, resulting in non-cell-autonomous reduced neurite outgrowth. Importantly, restoring *EAAT2* expression by Riluzole efficiently reverses the impaired synapse formation and function induced by *WFS1* deficiency in cerebral organoids and co-culture. Furthermore, Riluzole rescues the depressive-like behavior, the impaired recognition and spatial memory in *Wfs1* conditional knockout mice. Thus, our study provides mechanistic insights into psychiatric disorders of WS, and highlights the pathogenic role of *WFS1*-deficient astrocytes and proposes a potential therapeutic approach with Riluzole.

## Results

### *WFS1*-deficient cerebral organoids recapitulate progressively neuronal loss in WS

Since WS is a recessive disease caused by LOF mutations in *WFS1* gene, we applied CRISPR/Cas9 to introduce *WFS1* knock-out (*WFS1*^*−/−*^) mutations in three hESCs lines, including H1, H9 and HuES8, respectively (Fig. S1A). There was no obvious difference in cell morphology, self-renewal capacity and pluripotency, and the growth rate between *WFS1*^*−/−*^ and its wild-type counterpart (WT) hESCs (Fig. S1B–E). All three hESCs lines showed similar results (Fig. S1F).

**Fig. 1:**
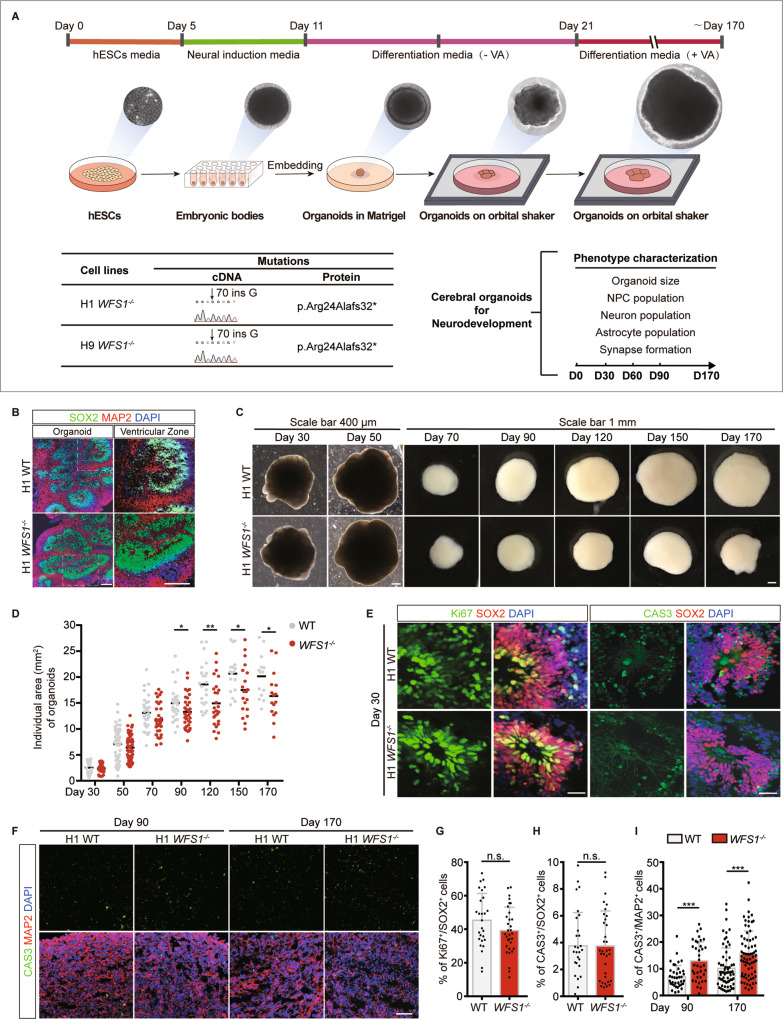
*WFS1* deficiency reduces organoid size and increases neuronal loss in cerebral organoids. **A** Schematic of the strategy of modeling WS neuropathy with cerebral organoids. **B** Representative immunostaining for SOX2 (green), MAP2 (red) and DAPI (blue) in WT and *WFS1*^*−/−*^ cerebral organoids at Day 50. Scale bar, 200 μm. Right panels are magnified views of ventricular zone (VZ) in the hollow box region of left panels. Scale bar, 200 μm. **C** Representative bright-field images of WT and *WFS1*^*−/−*^ cerebral organoids at Day 30, Day 50, Day 70, Day 90, Day 120, Day 150, Day 170. Scale bar, 400 μm (Day 30, 50). Scale bar, 1 mm (Day 70, 90, 120, 150, 170). **D** Quantification of the individual area (mm^2^) of WT and *WFS1*^*−/−*^ cerebral organoids, *n* ≥ 17 individual organoids. **E** Immunostaining for SOX2 (red), the proliferation marker Ki67 and the apoptotic marker cleaved caspase-3 (CAS3) (green), and DAPI (blue) in WT and *WFS1*^*−/−*^ cerebral organoids at Day 30. Scale bar, 20 μm. **F** Immunostaining for MAP2 (red), CAS3 (green) and DAPI (blue) in WT and *WFS1*^*−/−*^ cerebral organoids at Day 90 and Day 170. Scale bar, 50 μm. **G** Quantification of the percentage of Ki67^+^ cells among the total number of SOX2^+^ NPCs in WT and *WFS1*^*−/−*^ cerebral organoids at Day 30, *n* = 3 individual organoids. **H** Quantification of the percentage of CAS3^+^ cells among the total number of SOX2^+^ NPCs in WT and *WFS1*^*−/−*^ cerebral organoids at Day 30, *n* = 3 individual organoids. **I** Quantification of the percentage of CAS3^+^ cells among the total number of MAP2^+^ neurons in WT and *WFS1*^*−/−*^ cerebral organoids at Day 90 and Day 170, *n* = 3 individual organoids. Data are presented as mean ± SD. *p* values calculated by unpaired two-tailed Student’s *t* test were *p < 0.05, **p < 0.01, and ***p < 0.001.

**Fig. 2:**
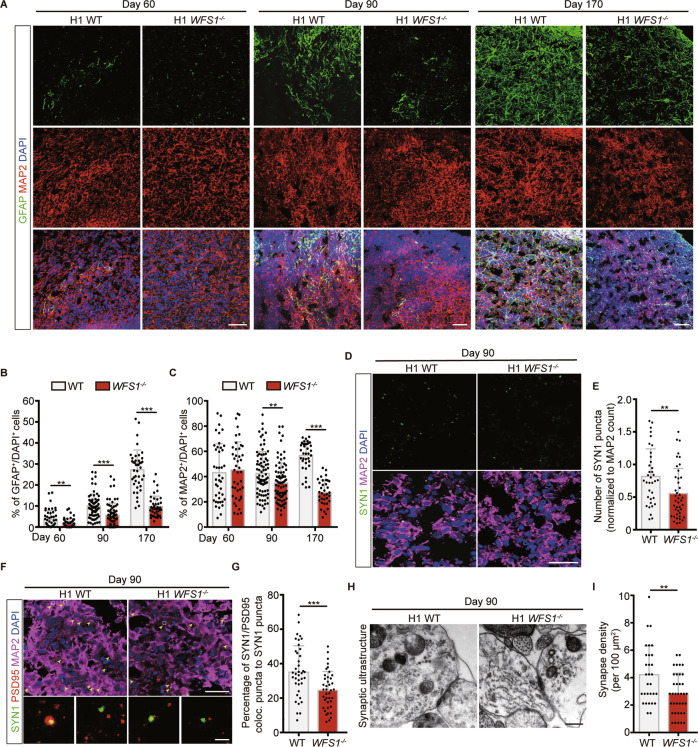
*WFS1* deficiency reduces astrocytes population and impairs synapse formation in cerebral organoids. **A** Immunostaining for GFAP (green), MAP2 (red) and DAPI (blue) in WT and *WFS1*^*−/−*^ cerebral organoids at Day 60, Day 90 and Day 170. Scale bar, 50 μm. **B**, **C** Quantification of the percentage of GFAP^+^ and MAP2^+^ cells among the total number of DAPI^+^ cells in WT and *WFS1*^*−/−*^ cerebral organoids at Day 60, Day 90 and Day 170, *n* ≥ 3 individual organoids. **D** Immunostaining for the presynaptic marker Synapsin 1 (SYN1) (green) and MAP2 (magenta) in WT and *WFS1*^*−/−*^ cerebral organoids at Day 90. Scale bar, 25 μm. **E** Quantification of the number of SYN1 puncta normalized to MAP2 count in WT and *WFS1*^*−/−*^ cerebral organoids at Day 90, *n* = 3 individual organoids. **F** Immunostaining for the SYN1 (green), postsynaptic marker PSD95 (red) and MAP2 (magenta) in WT and *WFS1*^*−/−*^ cerebral organoids at Day 90. Scale bar, 25 μm. Arrowheads indicate colocalization of SYN1 and PSD95. Lower panels are magnified views of the boxed region in the upper panel. Scale bar, 2 μm. **G** Quantification of the percentage of SYN1/PSD95 colocalized puncta to SYN1 puncta count in WT and *WFS1*^*−/−*^ cerebral organoids at Day 90, *n* = 3 individual organoids. **H** Representative images of electron microscopy of synaptic ultrastructure in WT and *WFS1*^*−/−*^ cerebral organoids at Day 90. Scale bar, 400 nm. **I** Quantification of the number of synapse structure normalized to area (100 μm^2^) in WT and *WFS1*^*−/−*^ cerebral organoids at Day 90, *n* = 3 individual organoids. Data are presented as mean ± SD. *p* values calculated by unpaired two-tailed Student’s *t* test were *p < 0.05, **p < 0.01, and ***p < 0.001.

Next, we generated cerebral organoids from WT and *WFS1*^*−/−*^ H1 and H9 hESCs (commonly used for cerebral organoids generation) in suspension culture containing cortical differentiation medium [36] (Fig. 1A). Indeed, both WT and *WFS1*^*−/−*^ organoids formed 3D neural tissues with ventricular zone (VZ)-like structure surrounded by a thick layer of neural progenitor cells (NPCs) (SOX2-positive cells) on Day 50 (Fig. 1B). As the organoids kept on growing, in organoids from H1 cell line, a reduction in size of *WFS1*^*−/−*^ organoids was observed from Day 90 to Day 170 (Fig. 1C, D). In organoids from H9, the *WFS1*^*−/−*^ organoids showed decreased size since Day 60 (Fig. S2A, B). These results recapitulate the reduced brain volume observed in WS patients [39, 40]. To explore the mechanism underlying the size reduction, we assessed proliferative capacity and apoptosis of NPCs at Day 30 by immunostaining of Ki67 and cleaved caspase-3 (CAS3), and they were not affected by *WFS1* deficiency in both H1 and H9 organoids (Figs. 1E, G and H; S2C–F). Next, we performed the immunostaining of SOX2 and neuronal marker MAP2 in cerebral organoids on Day 50, and quantified the layer thickness of the SOX2^+^ VZ-like zones and the number of rosettes in the whole cerebral organoid. We found that both of them were significantly decreased in *WFS1*-deficient cerebral organoids as compared to WT cerebral organoids (Fig. S3A–D). Further, reduction of neurons in *WFS1*^*−/−*^ cerebral organoids was observed by MAP2 immunostaining on Day 90, Day 170 and Day 210 but not on Day 60 (Figs. 2A, C; S2I–M). Concurrently, we examined the apoptosis of neurons by immunostaining of MAP2 and CAS3. *WFS1*^*−/−*^ organoids displayed a higher percentage of MAP2^+^CAS3^+^ cells at Day 90 and Day 170 (Fig. 1F, I). Together, these results suggest that neuronal loss may account for the reduction in organoid size at later stages of differentiation, and *WFS1*^*−/−*^ cerebral organoids recapitulate aspects of reduced brain volume and increased neuronal loss observed in the patients of WS neuropathy in a progressive manner [41, 42].

### *WFS1* deficiency reduces astrocytes population and impairs synapse formation in cerebral organoids

Astrocytes have been known as an essential cell type for neuronal survival and morphogenesis [30, 43]. During brain development, astrogenesis occurs following neurogenesis which could be recapitulated in the developing cerebral organoids (Fig. S3H). Astrocytes were observed as early as Day 60 and kept on increasing as cerebral organoids grew by immunostaining of astrocyte marker GFAP. The number of GFAP^+^ astrocytes was much lower in *WFS1*^*−/−*^ cerebral organoids on Day 60, Day 90 and Day 170 compared to WT organoids (Figs. 2A, B; S2G, H). Astrocytes has been shown to induce synapse formation in neurons. We performed the immunostaining for the presynaptic marker Synapsin 1 (SYN1), and found that the number of SYN1 puncta colocalized with MAP2 was significantly decreased in *WFS1*^*−/−*^ cerebral organoids (Fig. 2D, E). Concurrently, the percentage of SYN1/PSD95 colocalized puncta to SYN1 puncta was significantly decreased in *WFS1*^*−/−*^ cerebral organoids as compared to WT organoids (Fig. 2F–G). Additionally, the synaptic ultrastructure was examined by electron microscopy and results showed a decrease of synapse density in *WFS1*^*−/−*^ cerebral organoids (Fig. 2H, I). Further, to evaluate the effect of *WFS1* deficiency on synapse function, we performed whole-cell patch-clamp recording on neurons within cerebral organoids. We found that the frequency but not amplitude of spontaneous excitatory postsynaptic current (sEPSC) of neurons within *WFS1*-deficient cerebral organoids was significantly decreased, compared to WT cerebral organoids (Fig. 5G, H). Altogether, these results demonstrate that *WFS1* deficiency impairs synapse formation and function in cerebral organoids.

### *WFS1* deficiency in neurons autonomously results in delayed neuronal differentiation and impaired synapse formation

Since recapitulated clinical features are from cerebral organoids, which contained diverse neural cell types, the results might be a reflection of changes in different cell populations. To evaluate *WFS1*’s role in neurons and astrocytes, we differentiated WT and *WFS1*^*−/−*^ HuES8 into NPCs using previously reported 2D culture methods [18, 44], which could be maintained and subsequently differentiated into neurons and astrocytes separately.

**Fig. 3:**
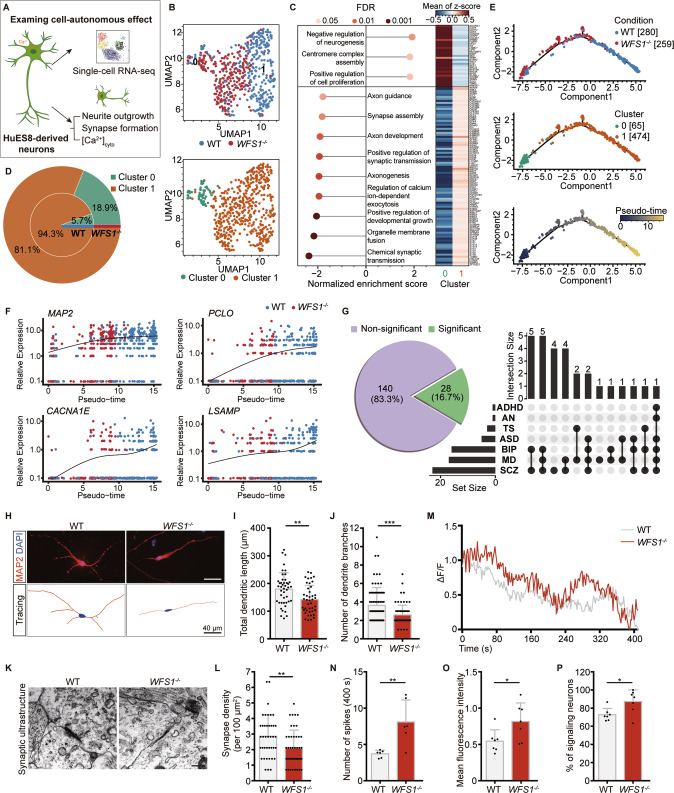
*WFS1* deficiency autonomously delays neuronal differentiation and impairs synapse formation. **A** Schematic of the strategy of investigating the cell-autonomous effect of *WFS1* deficiency in neurons. **B** UMAP plot of scRNA-seq data of 539 neurons that passed quality control. Each dot represents a single neuron. Cells are color coded for the corresponding conditions (WT or *WFS1*^*−/−*^, the top panel) and clusters (cluster 0 and 1, the bottom panel). **C** Dot plot showing significantly enriched GO terms of two clusters (left). GO terms with positive normalized enrichment scores (NES) mean they are enriched in cluster 0 and those with negative NESs mean they are enriched in cluster 1. And heatmap displaying the differentially expressed genes in the leading-edge genes of these terms (right). **D** Nested pie chart showing the difference in the proportion of cells in two clusters between WT and *WFS1*^*−/−*^ neurons. **E** Principal graph of constructed trajectory of WT and *WFS1*^*−/−*^neurons. Each dot represents a single neuron. Cells are color coded for the corresponding conditions (WT or *WFS1*^*−/−*^, the top panel), clusters (cluster 0 and 1, the middle panel) and inferred pseudo-time (the bottom panel). **F** Scatter plot of expression of genes over pseudo-time. MAP2, a marker gene of neuron. *PCLO*, *CACNA1E*, *LSAMP* are genes associated with psychiatric disorder. The black curve is the fitted curve, representing the trend of expression changes over pseudo-time. **G** Pie chart showing that among the genes related to psychiatric disorders, genes significantly correlated with pseudo-time account for 16.7% (28 out of 168). UpSet plot depicting overlap of genes which are both seven psychiatric disorders-associated and significantly pseudo-time correlated. ADHD attention-deficit/hyperactivity disorder, AN anorexia nervosa, TS Tourette syndrome, ASD autism spectrum disorder, BIP bipolar disorder, MD major depression, SCZ schizophrenia. **H** Immunostaining for MAP2 (red) and DAPI (blue) in WT and *WFS1*^*−/−*^ neurons. Scale bar, 40 μm. Lower panels are the corresponding tracings of WT and *WFS1*^*−/−*^ neurons. Scale bar, 40 μm. **I**, **J** Quantification of the total dendritic length (μm) and the number of dendrite branches, *n* = 4 independent experiments. **K** Representative images of electron microscopy of synaptic ultrastructure in WT and *WFS1*^*−/−*^ neurons. Scale bar, 400 nm. **L** Quantification of the number of synapse structure normalized to area (100 μm^2^) in WT and *WFS1*^*−/−*^ neurons, *n* = 3 independent experiments. **M** Representative singlecell traces of intracellular spontaneous calcium activity of WT and *WFS1*^*−/−*^ neurons. ΔF/F indicates the changes in fluorescence intensity reflecting cytosolic calcium activity in neurons. Intracellular spontaneous calcium activity analysis shown as calcium spike frequency (**N**), mean fluorescence intensity (**O**) and the percentage of signaling neurons (**P**) in WT and *WFS1*^*−/−*^ neurons, *n* = 6 fields from 3 independent experiments. Data are presented as mean ± SD. *p* values calculated by unpaired two-tailed Student’s *t* test were *p < 0.05, **p < 0.01, and ***p < 0.001.

To explore the cell-autonomous effect of *WFS1* deficiency on the transcriptome of neurons, we performed single-cell RNA sequencing (scRNA-seq) (Fig. 3A). Among total of 768 neurons, the scRNA-seq profiles of 539 neurons, including 280 neurons from WT NPCs and 259 neurons from *WFS1*^*−/−*^ NPCs, passed quality control (Fig S4A). The scRNA-seq profiles of neurons derived from *WFS1*^*−/−*^ NPCs looked generally different from those derived from WT NPCs, and all the 539 single neurons could be separated into two distinguishable clusters, cluster 0 and 1 (Fig. 3B). We carried out differential gene expression analysis using *edgeR-zingeR* [45, 46], and performed gene set enrichment analysis (GSEA). Among the GO terms enriched in cluster 0, we observed terms associated with negative regulation of differentiation and positive regulation of cell proliferation (Fig. 3C). By contrast, GO terms enriched in cluster 1 appeared to be associated with neuronal differentiation and synapse formation (Fig. 3C). So we speculated that the neurons in cluster 0 were less differentiated than those in cluster 1. Meanwhile, a significantly higher proportion of these less differentiated neurons were observed in the *WFS1*^*−/−*^ group (18.9%) than in the WT group (5.7%) (Fig. 3D). To further analyze the effects of *WFS1* deficiency on neuronal differentiation process, we constructed a trajectory for all WT and *WFS1*^*−/−*^ neurons using Monocle [47–49] and ordered the single neurons by the pseudo-time inferred for each of them. Consistent with the results of GSEA, cells in cluster 0, the less differentiated cluster, were positioned on the early stage of the trajectory and the *WFS1*^*−/−*^ neurons also intended to be on the left branch of the trajectory, suggesting *WFS1* deficiency delays neuronal differentiation (Fig. 3E). Along the trajectory, we identified 1,931 genes whose expression was significantly associated with the pseudo-time using Monocle (FDR ≤ 0.05). We observed the expression of *MAP2*, *PCLO*, *CACNA1E*, *LSAMP*, *ANKS1B*, *PPP2R2B*, *NEFM*, *GRIN2B*, *NRXN1* and *KCNB1* increased over pseudo-time and were lower in the *WFS1*^*−/−*^ group than in the WT group (Figs. 3F; S4B). These genes are known to be related with synapse formation and psychiatric disorders. Based on the above results, we carried out a systematic analysis to discover psychiatric disorders associated genes from the 1,931 genes. Specifically, we took the candidate genes of 146 linkage disequilibrium (LD)-independent SNPs predisposed to at least one psychiatric disorder reported by Cross-Disorder Group of the Psychiatric Genomics Consortium [50], and mapped them to 168 genes, which were considered as psychiatric disorders-associated genes. Among these genes, 28 showed significantly correlated expression with the pseudo-time, accounting for 16.7% (left panel of Fig. 3G), and most of these were reported to be associated with more than one mental disease (right panel of Fig. 3G). These results indicate that *WFS1* deficiency leads to delayed differentiation of neurons and affects the expression of genes associated with synapse formation and common psychiatric disorders.

To validate the scRNA-seq analysis, neurons were stained with SOX2 and evaluated by intracellular flow cytometric analysis, to measure the population of NPCs in neuron cultures. Flow cytometry analysis showed the percentage of SOX2^+^ cells significantly increased in *WFS1*^*−/−*^ neurons compared to WT neurons (Fig. S4E, F). Meanwhile, the percentage of differentiated neurons in the 3-week neuron cultures was quantified by magnetic-activated cell sorting for CD44^-^CD184^-^ cells [51]. We found that the proportion of CD44^-^CD184^-^ cells was significantly lower in *WFS1*^*−/−*^ neurons compared to WT neurons (Fig. S4G). Further, we performed the immunostaining for SOX2, MAP2 and GFAP simultaneously in cerebral organoids and found that the number of SOX2^+^ cells was much higher in *WFS1*^*−/−*^ cerebral organoids on Day 90 and Day 170 as compared to WT cerebral organoids (Fig. S3E, F). On Day 90 and Day 170, the majority of SOX2^+^ cells were SOX2-expressing astrocytes rather than NPCs [52]. To exclude this possibility, we then quantified the proportion of SOX2^+^GFAP^-^MAP2^-^ cells more precisely to represent NPCs population. And we found that the percentage of SOX2^+^GFAP^-^MAP2^-^ cells was significantly higher in *WFS1*^*−/−*^ cerebral organoids on Day 90 and Day 170 as compared to WT cerebral organoids (Fig. S3G). Together, these results suggest that neuronal differentiation is delayed by *WFS1* deficiency.

To validate the impaired synapse formation indicated by scRNA-seq data analysis, we examined neurite outgrowth measured by MAP2 staining in neurons. *WFS1*^*−/−*^ neurons displayed significantly reduced total dendritic length and decreased number of dendrite branches compared with WT neurons (Fig. 3H–J). We also observed increased neuronal cell death in *WFS1*^*−/−*^ neurons as compared with WT neurons (Fig. S4C, D). As expected, we observed decreased synapse density in *WFS1*^*−/−*^ neurons as measured by electron microscopy compared to WT neurons (Fig. 3K, L). Moreover, it has been reported that *WFS1* regulates calcium storage within ER, and its deficiency results in dysfunction of ER and consequent elevation of cytosolic Ca^2+^ ([Ca^2+^]_cyto_) [28, 53]. To further identify the role of disturbed cytosolic Ca^2+^ in regulation of neurite outgrowth resulted from *WFS1* deficiency, we conducted a time-lapse recording of spontaneous cytosolic Ca^2+^ activity in neurons loaded with Fluo-4/AM. Spontaneous cytosolic Ca^2+^ activity as measured by changes in fluorescence intensity (ΔF/F) demonstrated that there was an increase in both frequency and amplitude of cytosolic Ca^2+^ activity in *WFS1*^*−/−*^ neurons as compared to WT neurons (Fig. 3M–O). There was also an increase in the percentage of signaling neurons showing spontaneous Ca^2+^ transients in *WFS1*^*−/−*^ neurons (Fig. 3P). To investigate whether the elevation of cytosolic Ca^2+^ contributes to impaired neurite outgrowth, we treated WT neurons with 0.125 μM Thapsigargin, an inhibitor for sarco/endoplasmic reticulum calcium ATPase (SERCA) pump, to increase cytosolic calcium levels. After 24 h treatment, increased spontaneous cytosolic Ca^2+^ activity resulted in reduced total dendritic length and the number of dendrite branches in WT neurons treated with Thapsigargin group as compared to control group (Fig. S5A–G). Previous studies have shown that Dantrolene is an inhibitor of the ryanodine receptors and suppresses calcium leakage from the ER to cytosol, lowering cytosolic calcium level [54, 55]. Thus, we treated *WFS1*^*−/−*^ neurons with 8 μM Dantrolene for 48 h and found that the cytosolic Ca^2+^ activity was significantly decreased in *WFS1*^*−/−*^ neurons (Fig. S5H–K); the total dendritic length and the number of dendrite branches in *WFS1*^*−/−*^ neurons were also significantly rescued by Dantrolene treatment as compared to control (Fig. S5L–N). Altogether, these results suggest that cell-autonomous effect of *WFS1* deficiency in neurons contributes to the impaired synapse formation, which is induced by elevation of cytosolic Ca^2+^.

### *WFS1* deficiency renders astrocytes toxic to neurite outgrowth through excessive glutamate in a non-cell-autonomous manner

**Fig. 4:**
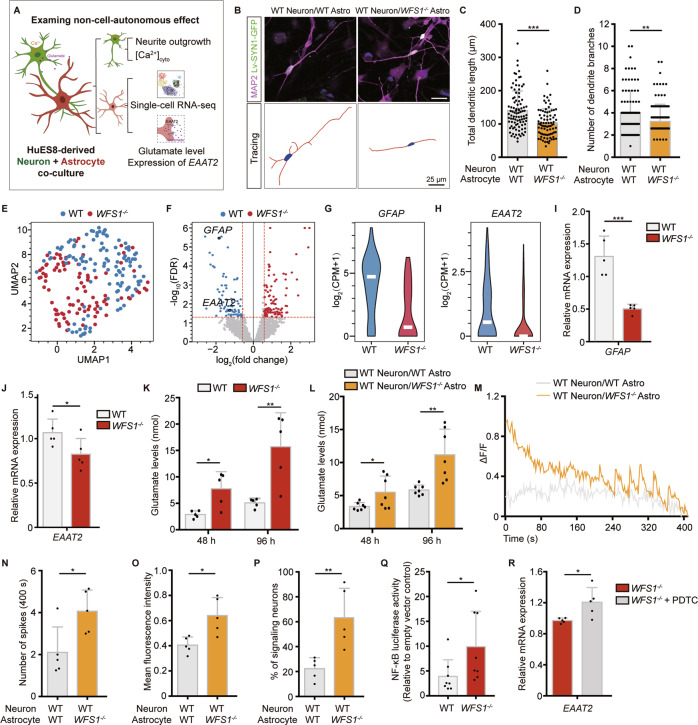
Astrocytic *WFS1* deficiency impairs neurite outgrowth in non-cell-autonomous manner. **A** Schematic of the strategy of investigating the non-cell-autonomous effect of *WFS1*^*−/−*^astrocytes. **B** Representative images of co-culture of WT/*WFS1*^*−/−*^ astrocytes and WT neurons, neurons labeled with lentivirus SYN1::GFP were stained with MAP2 (magenta). Scale bar, 25 μm. Lower panels are the corresponding tracings of neurons. Scale bar, 25 μm. **C**, **D** Quantification of the total dendritic length (μm) and the number of dendrite branches, *n* = 3 independent experiments. **E** UMAP plot of scRNA-seq data of 207 astrocytes that passed quality control. Each dot represents a single cell. Cells are color coded for the corresponding conditions (WT or *WFS1*^*−/−*^). **F** Volcano plot depicting differential gene analysis between WT and *WFS1*^*−/−*^ astrocytes. Dots of color indicate differentially expressed genes, that is, those with |log_2_FC | > 0.58 and FDR ≤ 0.05 (red for up- and blue for down-regulated genes in *WFS1*^*−/−*^ astrocytes). **G**, **H** Violin plots displaying the expression of *GFAP* and *EAAT2* in WT and *WFS1*^*−/−*^ astrocytes. The y-axis shows the log-transformed normalized read count. **I**, **J** Quantitative real-time PCR analysis of *GFAP* and *EAAT2* in WT and *WFS1*^*−/−*^ astrocytes, *n* = 5 independent experiments. **K** Glutamate levels (nmol) at 48 h and 96 h in WT and *WFS1*^*−/−*^ astrocytes culture, *n* = 5 independent experiments. **L** Glutamate levels (nmol) at 48 h and 96 h in co-culture of WT neurons and WT/*WFS1*^*−/−*^ astrocytes, *n* = 7 independent experiments. **M** Representative single cell traces of intracellular spontaneous calcium activity of WT neurons co-cultured with WT/*WFS1*^*−/−*^ astrocytes. Intracellular spontaneous calcium activity analysis shown as calcium spike frequency (**N**), mean fluorescence intensity (**O**) and the percentage of signaling neurons (**P**) in WT neurons co-cultured with WT/*WFS1*^*−/−*^ astrocytes, *n* = 5 fields from 3 independent experiments. **Q** NF-κB luciferase activity in WT and *WFS1*^*−/−*^ astrocytes, relative to empty vector control group, *n* = 8 independent experiments. **R** Quantitative real-time PCR analysis of *EAAT2* in *WFS1*^*−/−*^ astrocytes and NF-κB inhibitor PDTC treated-*WFS1*^*−/−*^ astrocytes, *n* = 5 independent experiments. Data are presented as mean ± SD. *p* values calculated by unpaired two-tailed Student’s *t* test were *p < 0.05, **p < 0.01, and ***p < 0.001.

In many neurological disorders, disease-relevant astrocytes confer detrimental effects on neurons resulting in reduced neurite outgrowth and synapse formation [31, 33]. However, *WFS1*’s function in interplays between astrocytes and neurons remain elusive. To investigate the non-cell-autonomous effect of astrocytes with *WFS1* deficiency on neurons, neuron-astrocyte co-culture was applied as a simplified model as previously reported [56] (Figs. 4A and S6B). We found that there was a significant decrease in neurite outgrowth including total dendritic length and the number of dendrite branches in WT neurons with *WFS1*^*−/−*^ astrocytes group (WT neuron/*WFS1*^*−/−*^ astro) compared with WT neurons with WT astrocytes group (WT neuron/WT astro) (Fig. 4B–D). And neuronal loss within neuron-astrocyte co-culture was also increased in WT neuron/*WFS1*^*−/−*^ astro group (Fig. S6C, D). Accordingly, these results suggest that *WFS1* deficiency in astrocytes plays an essential pathogenic role in neurite outgrowth with a non-cell-autonomous manner.

To identify the possible causes underlying the detrimental effects of *WFS1*-deficient astrocytes on neurons, we performed scRNA-seq of WT and *WFS1*^*−/−*^ astrocytes. The scRNA-seq profiles of 207 single astrocytes passed quality control and were retained for further analysis (Fig. S6A). At first glance, there was no obvious clusters among these cells (Fig. 4E). Genes with log_2_FC > 0.58 and FDR ≤ 0.05 were identified as differentially expressed genes (DEGs), including 100 up-regulated and 73 down-regulated genes in *WFS1*^*−/−*^ group compared with WT group (Fig. 4F). Among them, *GFAP* was significantly down-regulated in *WFS1*^*−/−*^ group (Fig. 4G). Moreover, *EAAT2*, which encodes glutamate transporter was also downregulated in *WFS1*^*−/−*^ group (Fig. 4H). Altogether, these results indicate that the expression of *GFAP* and transportation of glutamate in *WFS1*-deficient astrocytes might be affected.

We performed q-PCR to empirically validate the decreased gene expression of *GFAP* in *WFS1*^*−/−*^ astrocytes (Fig. 4I), which was in line with the reduced GFAP^+^ astrocytes population in cerebral organoids. *EAAT2* is a transporter mainly expressed in astrocytes for glutamate transportation which is essential for maintaining glutamate homeostasis [57, 58]. The decreased *EAAT2* level revealed by the scRNA-seq data was also validated by q-PCR (Fig. 4J). Next, glutamate assay was performed to assess extracellular glutamate level in astrocytes culture. There was a dramatic increase of the glutamate level in medium at 48 h and 96 h of *WFS1*^*−/−*^ astrocytes as compared to WT astrocytes (Fig. 4K), suggesting excessive glutamate is induced by *WFS1*-deficient astrocytes with *EAAT2* downregulation. To further confirm this, we also analyzed the glutamate level in neuron-astrocyte co-culture. Glutamate level was significantly higher in WT neuron/*WFS1*^*−/−*^ astro group as compared to WT neuron/WT astro group (Fig. 4L). To examine the detrimental effect of excessive glutamate on neurite outgrowth, WT neurons were treated with 100 μM glutamate (close to the glutamate level monitored in medium of *WFS1*-deficient astrocytes) for 48 h. There was a decrease in total dendritic length, the number of dendrite branches and the percentage of MAP2^+^ neurons in glutamate-treated neurons as compared to control group (Fig. S7A–E). Accordingly, these results suggest that excessive glutamate elicited by *WFS1*^*−/−*^ astrocytes with downregulation of *EAAT2* expression leads to impaired neurite outgrowth in neurons.

Furthermore, it has been reported that excitotoxicity elicited by excessive glutamate induces calcium influx into neurons and elevates cytosolic calcium in these cells, which results in impaired neurite outgrowth and synapse formation [59–62]. Thus, we measured the spontaneous cytosolic Ca^2+^ activity in WT neurons co-cultured with WT/*WFS1*^*−/−*^ astrocytes loaded with Rhod-4/AM. As expected, changes in fluorescence intensity (ΔF/F) showed that there was indeed an increase in the Ca^2+^ activity frequency, amplitude and percentage of signaling neurons in WT neuron/*WFS1*^*−/−*^ astro group compared with WT neuron/WT astro group (Fig. 4M–P). Additionally, WT neurons treated with 100 μM glutamate also exhibited increased cytosolic Ca^2+^ activity (Fig. S7F–I). These results suggest that *WFS1* deficiency renders astrocytes toxic to neurons by elevating neuronal cytosolic calcium *via* excessive glutamate. To note, our results demonstrate that *WFS1* deficiency also autonomously elevates cytosolic calcium in neurons with impaired neurite outgrowth. Collectively, these results suggest that cell-autonomous and non-cell-autonomous detrimental effects induced by *WFS1* deficiency in neurons and astrocytes converge on increased neuronal cytosolic calcium pathway (Fig. 6S).

Several studies have shown that NF-κB pathway is critical for the transcriptional regulation of *EAAT2*, as *EAAT2* has a binding site in its promotor for NF-κB [63–66]. To further investigate the mechanisms underlying downregulation of *EAAT2* transcriptional level, we explored whether NF-κB pathway is involved in the regulation of *EAAT2* mRNA levels under the situation of *WFS1* deficiency. We examined the NF-κB activity by immunostaining of P50 and P65 nuclear translocation and luciferase reporter assay in differentiated astrocytes. Both the intensity of P50 and P65 in the nuclei area and the NF-κB luciferase activity were significantly increased in *WFS1*^*−/−*^ astrocytes as compared to WT astrocytes (Figs. 4Q; S6E–G). To further identify the role of NF-κB activation in regulation of *EAAT2* mRNA levels, we applied 100 μM PDTC, a pharmacological inhibitor of NF-κB, into *WFS1*^*−/−*^ astrocytes. After 40 min treatment, the mRNA levels of *EAAT2* were significantly reversed and NF-κB activation was suppressed in PDTC*-*treated *WFS1*^*−/−*^ astrocytes, as compared to control group (Figs. 4R; S6H). These results suggest that the activation of NF-κB indeed results in the decreased mRNA level of *EAAT2*. *WFS1* deficiency leads to high level of endoplasmic reticulum (ER) stress [42], and enhanced ER stress could activate NF-κB pathway [67]. To investigate whether *WFS1* deficiency leads to NF-κB activation by upregulating ER stress, we examined the expression of three major components for sensing ER stress, *PERK*, *ATF6* and *IRE*, by q-PCR. We found that the mRNA expression of *PERK* and *ATF6* were significantly increased in *WFS1*^*−/−*^ astrocytes as compared to WT astrocytes (Fig. S6I), suggesting that the ER stress is upregulated in *WFS1*^*−/−*^ astrocytes. And then we treated WT astrocytes with 0.125 μM Thapsigargin, an ER stress inducer by increasing cytosolic calcium levels. After 24 h treatment, the NF-κB luciferase activity was significantly increased as compared to control group (Fig. S6J), indicating that upregulated ER stress induced by *WFS1* deficiency leads to NF-κB activation. Altogether, these results suggest that activation of NF-κB contributes to downregulation of *EAAT2* transcriptional level, which is induced by enhanced ER stress in *WFS1*-deficient astrocytes.

### Riluzole reverses impaired synapse formation induced by *WFS1* deficiency

**Fig. 5:**
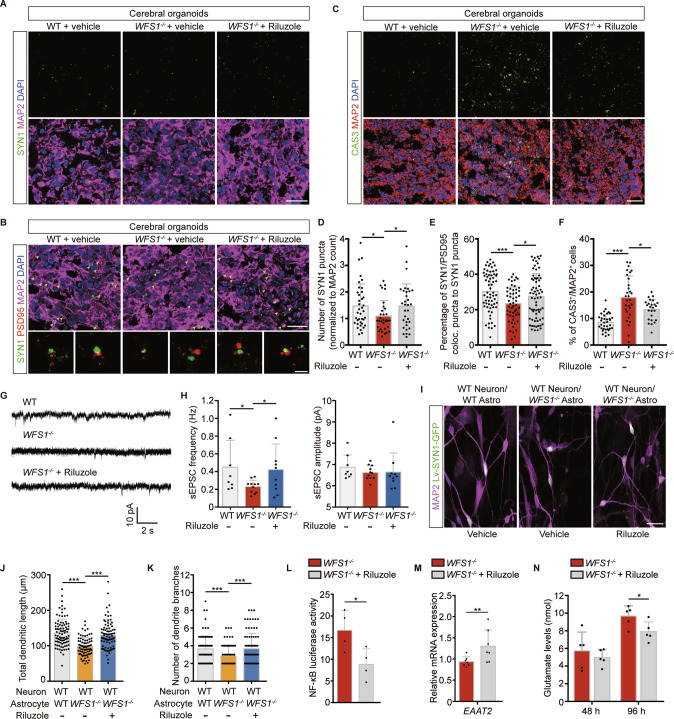
Impaired synapse formation and function induced by *WFS1* deficiency is reversed by Riluzole. **A** Immunostaining for the SYN1 (green) and MAP2 (magenta) in WT and *WFS1*^*−/−*^ cerebral organoids treated with 5 μM Riluzole or vehicle at Day 90. Scale bar, 25 μm. **B** Immunostaining for the SYN1 (green), PSD95 (red) and MAP2 (magenta) in WT and *WFS1*^*−/−*^ cerebral organoids treated with 5 μM Riluzole or vehicle at Day 90. Scale bar, 25 μm. Arrowheads indicate colocalization of SYN1 and PSD95. Lower panels are magnified views of the boxed region in the upper panel. Scale bar, 2 μm. **C** Immunostaining for MAP2 (red), CAS3 (green) and DAPI (blue) in WT and *WFS1*^*−/−*^ cerebral organoids treated with 5 μM Riluzole or vehicle at Day 90. Scale bar, 50 μm. **D** Quantification of the number of SYN1 puncta normalized to MAP2 count in WT and *WFS1*^*−/−*^ cerebral organoids treated with 5 μM Riluzole or vehicle at Day 90, *n* = 3 individual organoids. **E** Quantification of the percentage of SYN1/PSD95 colocalized puncta normalized to SYN1 puncta count in WT and *WFS1*^*−/−*^ cerebral organoids treated with 5 μM Riluzole or vehicle at Day 90, *n* = 3 individual organoids. **F** Quantification of the percentage of CAS3^+^ cells among the total number of MAP2^+^ neurons in WT and *WFS1*^*−/−*^ cerebral organoids treated with 5 μM Riluzole or vehicle at Day 90, *n* = 3 individual organoids. **G** Representative traces of sEPSC of WT, *WFS1*^*−/−*^ and Riluzole-treated *WFS1*^*−/−*^ cerebral organoids. **H** Quantification of the frequency and amplitude of sEPSC for WT, *WFS1*^*−/−*^ and Riluzole-treated *WFS1*^*−/−*^ cerebral organoids, *n* = 3 individual organoids. **I** Representative images of co-culture of WT neurons and WT/*WFS1*^*−/−*^ astrocytes treated with 5 μM Riluzole or vehicle, neurons labeled with lentivirus SYN1::GFP were stained with MAP2 (magenta). Scale bar, 25 μm. **J**, **K** Quantification of the total dendritic length (μm) and the number of dendrite branches, *n* = 3 independent experiments. **L** NF-κB luciferase activity in *WFS1*^*−/−*^ astrocytes treated with 5 μM Riluzole or vehicle, *n* = 4 independent experiments. **M** Quantitative real-time PCR analysis of *EAAT2* in *WFS1*^*−/−*^ astrocytes treated with 5 μM Riluzole or vehicle, *n* = 7 independent experiments. **N** Glutamate levels (nmol) at 48 h and 96 h in *WFS1*^*−/−*^ astrocytes culture treated with 5 μM Riluzole or vehicle, *n* = 5 independent experiments. Data are presented as mean ± SD. *p* values calculated by unpaired two-tailed Student’s *t* test were *p < 0.05, **p < 0.01, and ***p < 0.001.

It is known that Riluzole upregulates astrocytic *EAAT2* expression to promote glutamate clearance [68, 69]. To investigate whether Riluzole could be used to treat impaired synapse formation in WS, we treated cerebral organoids on Day 90 with 5 μM Riluzole or vehicle for 7 days, respectively. We found that the number of SYN1 puncta significantly increased in Riluzole-treated cerebral organoids as compared to control group (Fig. 5A, D). Furthermore, Riluzole significantly increased the percentage of SYN1/PSD95 colocalized puncta to SYN1 puncta in *WFS1*^*−/−*^ cerebral organoids compared with control group (Fig. 5B, E). Concurrently, the apoptosis of neurons in *WFS1*^*−/−*^ cerebral organoids were significantly reduced by Riluzole as compared to control group (Fig. 5C, F). To examine the rescue effect of Riluzole on the electrophysiological properties of neurons, cerebral organoids were treated with 5 μM Riluzole since Day 50, then whole-cell patch-clamp recording was performed until Day 150. We found that the decreased frequency of sEPSCs was significantly rescued in Riluzole-treated cerebral organoids as compared to control in *WFS1*-deficient cerebral organoids (Fig. 5G, H). Next, we examined the effect of Riluzole on neurite outgrowth defects induced by *WFS1*-deficient astrocytes. We treated WT neuron co-cultured with *WFS1*^*−/−*^ astrocytes with 5 μM Riluzole or vehicle for 48 h, respectively. As compared to WT neuron co-cultured with *WFS1*^*−/−*^ astrocytes group, Riluzole almost fully reversed the defects in total dendritic length and the number of dendrite branches (Fig. 5I–K) and reduced the neuronal loss (Fig. S7J, K). Furthermore, Riluzole treatment decreased the NF-κB luciferase activity, restored astrocytic *EAAT2* expression and decreased excessive glutamate level in *WFS1*^*−/−*^ astrocytes (Fig. 5L–N). Taken together, these results suggest that Riluzole could rescue disrupted synapse formation and function, as well as neuronal loss induced by *WFS1* deficiency.

### Riluzole reverses behavioral defects in *Wfs1* conditional knockout mice

**Fig. 6:**
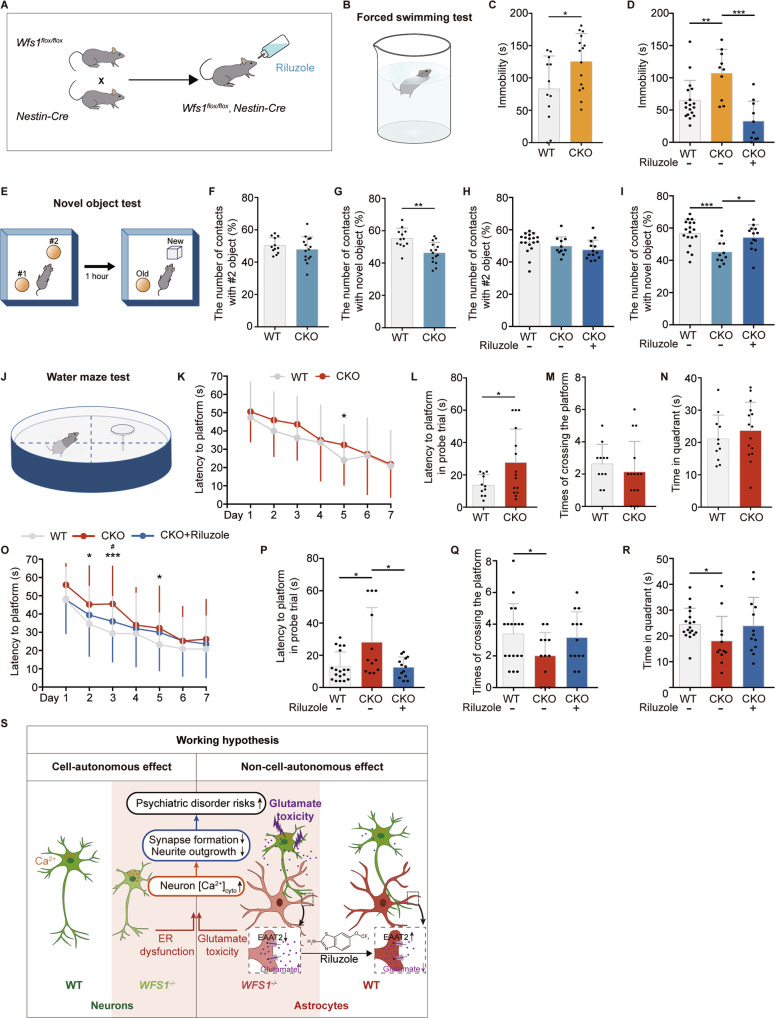
Riluzole reverses behavioral defects in *Wfs1* conditional knockout mice. **A** Schematic of the strategy of generating *Wfs1* conditional knockout mice for Riluzole treatment. **B** Schematic of the forced swimming test. **C** Analysis of the immobility time during the forced swimming test in WT and CKO mice, *n* ≥ 12. **D** Analysis of the immobility time during the forced swimming test after Riluzole treatment in WT and CKO mice, *n* ≥ 11. **E** Schematic of the novel object test. **F**, **G** Quantification of the percentage of the number of contacts with #2 or novel object in WT and CKO mice during acquisition and recognition session, *n* ≥ 12. **H**, **I** Quantification of the percentage of the number of contacts with #2 or novel object in WT and CKO mice during acquisition and recognition session after Riluzole treatment, *n* ≥ 11. **J** Schematic of the water maze test. **K** Latency to find the platform in WT and CKO mice during 7-day training sessions, *n* ≥ 12. On the probe trial day, quantification of the latency to the original position of the platform (**L**), times of crossing the platform area (**M**) and the time spent in quadrant of the platform (**N**) in WT and CKO mice, *n* ≥ 12. **O** Latency to find the platform in WT and CKO mice during 7-day training sessions after Riluzole treatment, *n* ≥ 11. On the probe trial day, quantification of the latency to the original position of the platform (**P**), times of crossing the platform area (**Q**) and the time spent in quadrant of the platform (**R**) in WT and CKO mice after Riluzole treatment, *n* ≥ 11. **S** The working hypothesis underlying the autonomous effect by *WFS1* deficiency in neurons and non-autonomous effect by astrocytic *WFS1* deficiency. Data are presented as mean ± SD. *p* values calculated by unpaired two-tailed Student’s *t* test were *p < 0.05, **p < 0.01, and ***p < 0.001. Two-way ANOVA was used for analysis of latency during 7-day training sessions after Riluzole treatment in the water maze test, *p < 0.05, **p < 0.01, and ***p < 0.001 for comparison of WT and CKO mice, ^#^*p* < 0.05 for comparison of the effect of Riluzole treatment.

Disrupted synapse formation and function underlies many psychiatric disorders, and patients with WS have been reported that suffering from a variety of psychiatric disorders including depression, impairments of recognition performance and memory [6, 70–73]. To recapitulate the psychiatric disturbances in patients, we conducted a series of behavioral studies including forced swimming test (depression), novel object test (recognition memory) and water maze test (spatial memory) in the *Wfs1* conditional knockout mice (CKO mice). We generated the conditional *Wfs1* knockout mice by crossing the *Wfs1-*flox mice (*Wfs1*^*flox/flox*^) with *Nestin-Cre* transgenic mice (Fig. 6A). The *Nestin-Cre* mice expressed Cre recombinase under the control of the *Nestin* promotor, thus *Wfs1* would be knocked out in neuroepithelial cells as they convert to radial progenitors and initiate neurogenesis, which is in line with our in vitro system.

In the forced swimming test, CKO mice showed a significant increase in immobility time compared to WT mice, indicating that mice developed depressive-like behavior induced by *Wfs1* deficiency (Fig. 6B, C). In the novel object test, both WT and CKO mice showed the similar number of contacts with the two objects in the session of acquisition (having two same objects) (Fig. 6E, F). However, in the session of recognition (having a familiar and a novel object), WT but not CKO mice showed significantly more contacts with the novel object than the familiar one (Fig. 6G), suggesting that CKO mice exhibit deficits in recognition memory. In the water maze test, CKO mice showed longer latency to find the platform during the 7-day training sessions, significant difference especially appeared on Day 5 (Fig. 6J, K). On the probe trial day, platform was removed and mice were allowed to swim for 60 s. Latency to the original platform position was significantly increased in CKO mice as compared to WT mice (Fig. 6L). There was no significant difference in the times of crossing the platform and time in the quadrant of platform in CKO mice compared to WT mice (Fig. 6M, N). These data suggest that *Wfs1* deficiency could impair the spatial memory.

To further identify the rescue effect of Riluzole on these behavioral defects, we treated CKO mice with Riluzole at the concentration of 50 mg/kg/day in the drinking water from the age of 3 weeks for 2 months (this dose was previously tested in mice [74]). Riluzole treatment mice exhibited reversed increased immobility in the forced swimming test (Fig. 6D); more contacts with the novel object in the novel object test (Fig. 6H, I); shorter latency to the platform during the training sessions, which was significantly different on Day 3 in the water maze test (Fig. 6O); decreased latency to the platform on the probe trial (Fig. 6P) and an increased trend in the times of crossing the platform and time in the quadrant of platform compared to control in CKO mice (Fig. 6Q, R). These results demonstrate that Riluzole could reverse most of the behavioral defects in *Wfs1*-deficient mice. Overall, Riluzole could be potentially applied to treat both disrupted synapse formation and function, as well as neuronal loss in WS patients.

## Discussion

The key to investigation of WS relies on proper human models, which is limited by ethic issue and scarcity of human samples. The effect of *WFS1* loss of function on neuronal dysfunction has been modeled in rodents and *Drosophila* [27, 75, 76]. However, these models do not adequately recapitulate defects by *WFS1* deficiency in human brain development, especially in investigating its role in mental illness. In this study, we applied a multi-dimensional strategy of combining 3D cerebral organoids and 2D neural differentiation derived from hESCs to illuminate the role of the causative gene *WFS1* in psychiatric disorders underlying WS. We found that *WFS1*-deficient cerebral organoids not only phenocopied progressive neuronal loss in WS patients, but also showed impaired synapse formation which underlies common psychiatric disorders, providing a defective structural basis for WS.

Synapse is the basic structure element for neural circuit. Recent studies reveal that impaired synaptogenesis and synaptic dysfunction contribute to psychiatric disorders. Moreover, variants of numerous genes involved in synapse formation during brain neurodevelopment have been identified as genetic risk factors for psychiatric disorders by genome-wide association study (GWAS) [50, 77]. To further elucidate the pathogenesis, recent technical breakthroughs with pluripotent hESCs derived neural cells (2D) and cerebral organoids (3D) are applied to model psychiatric disorders possessing genetic predispositions [23, 24, 78, 79]. Here, from genotype to phenotype, by applying 3D cerebral organoids and 2D neural cells derived from hESCs, our results revealed that *WFS1* deficiency resulted in reduced neurite outgrowth and consequent impaired synapse formation and function, which might explain the psychiatric symptoms observed in the WS patients. Nevertheless, further questions remain to be explored. Neurite outgrowth is a process of neurite extension. How *WFS1* as an ER stress regulator controls this process and regulates the precise neuronal connectivity is unclear.

Astrocytes are essential for synapse formation and maintenance, and recent studies reveal that malfunction of astrocytes underlie psychiatric disorders, such as schizophrenia and autism [80, 81]. These facts lead us to further explore the interplay between neurons and astrocytes affected by *WFS1* deficiency in neurite outgrowth and consequent synapse formation. The complexity was unraveled by 2D neural differentiation to induce neurons and astrocytes separately. On one hand, we found that *WFS1* deficiency autonomously elevated cytosolic calcium and reduced neurite outgrowth, contributing to impaired synapse formation. On the other hand, *WFS1*-deficient astrocytes elicited reduced neurite outgrowth in non-cell-autonomous manner, highlighting the essentiality of astrocytes. Mechanistically, we found that *WFS1* deficiency decreased the expression of the *EAAT2* in astrocytes by NF-κB activation, resulting in excessive glutamate and consequent elevated cytosolic calcium and reduced neurite outgrowth in neurons. More specifically, how NF-κB regulates *EAAT2* in astrocytes to control glutamate level warrants investigation. Further exploration should be focused on examining the underlying molecular mechanism.

Unfortunately, there is no effective therapy to treat WS which is hampered by limited understanding of the pathogenesis. It has been reported that Riluzole acts as a modulator to enhance *EAAT2* expression level and activity in astrocytes for glutamate clearance [68, 82, 83]. Thus, we explored the therapeutic effect of Riluzole and found that Riluzole treatment reversed the phenotypes of reduced neurite outgrowth, impaired synapse formation and function, and neuronal loss. Furthermore, Riluzole rescued the depressive-like behavior, the impaired recognition and spatial memory in *Wfs1* conditional knockout mice. Based on our discoveries, it will be highly valuable to test the therapeutic potential of Riluzole to treat WS neuropathy in future clinical studies.

## Materials and methods

### Generation of cerebral organoids

Cerebral organoids were generated as previously described [36]. Briefly, hESCs (H1 or H9) were dissociated to generate single cells. 9,000 cells were seeded in each well of an ultra-low-attachment 96-well plate (Corning) in mTeSR1 medium to form EBs. And then mTeSR1 was replaced with neural induction medium (NI medium) on Day 5 containing DMEM/F12 (Gibco™), 1× N2-supplement (Gibco™), 1× GlutaMAX (Gibco™), 1× MEM-NEAA (Sigma), 1× Penicillin/Streptomycin (Gibco™) and 1 μg/ml Heparin (Sigma). On Day 11, EBs were embedded in the center of the droplets of Matrigel (Corning), and then cultured in a 10-cm dish containing differentiation medium without vitamin A (1:1 mixture of DMEM/F12 and Neurobasal™ (Gibco™) medium containing 0.5× N2-supplement, 1× B27-supplement without vitamin A (Gibco™), 1× GlutaMAX, 0.5× MEM-NEAA, 1× Penicillin/Streptomycin, 1:4000 insulin (Sigma), 1 mg/mL NaHCO_3_). On Day 16, the EB droplets were transferred to an orbital shaker (57 r.p.m.). On Day 21, medium was replaced with differentiation medium with vitamin A same as above except B27-supplement without vitamin A replaced with 1× B27-Supplement (Gibco™), and 70 μg/mL vitamin C (Sigma) was added. On Day 40 onward, 1-2% Matrigel was added to differentiation medium with vitamin A. Medium was changed twice weekly until ready for further analysis.

### NPC induction and neuronal differentiation

HuES8 were maintained in mTeSR1 medium for 3 days, after that medium was changed to N2 medium (DMEM/F12 supplemented with 0.5× N2-Supplement) with 1 μM dorsomorphin and 1 μM SB431542 for 2 days. hESCs colonies were lifted off and cultured in suspension as embryoid bodies for 1 week within N2 medium on the orbital shaker (95 r.p.m.). Embryoid bodies then were mechanically dissociated, and plated on 10 μg/mL poly-L-ornithine (Sigma) and laminin (Gibco™)-coated dishes, and cultured in N/B medium (DMEM/F12 supplemented with 0.5× N2-Supplement, 0.5× B27-Supplement) with 20 ng/mL bFGF (Stemcell Technologies). Formed neural rosettes were manually isolated. To differentiate NPCs into neurons, bFGF was withdrawn from medium. After that N/B medium was changed every other day.

### Statistical analysis

The exact sample size for each experimental group were given in the figure legends. For cerebral organoids experiments, at least 3 individual organoids were collected for each experiment. For animal experiments, at least 11 animals were collected for each experiment. For other experiments, at least 3 independent experiments were performed for each experiment. The sample sizes were chosen based on our previous works and sufficient to confirm the conclusion, and no statistical methods were used to determine sample size. Investigator were blinded to the groups when assessing the outcomes. All data values were presented as mean ± SD. To compare the means of two normally distributed groups, unpaired two-tailed Student’s *t*-test was used. Two-way ANOVA followed by Tukey’s post hoc test was used with treatment and time as independent factors. Two-way ANOVA followed by Sidak’s post hoc test was used with the genotype and time as independent factors. The variance between the groups is similar. *p* values were *p < 0.05, **p < 0.01, and ***p < 0.001. All graphs were generated and analyzed using GraphPad Software Prism 7.

## Supplementary information

Supplemental information

Supplementary figure 1

Supplementary figure 2

Supplementary figure 3

Supplementary figure 4

Supplementary figure 5

Supplementary figure 6

Supplementary figure 7
