# Supplementary material for: Modeling disrupted synapse formation in wolfram syndrome using hESCs-derived neural cells and cerebral organoids identifies Riluzole as a therapeutic molecule
Source: Mol Psychiatry. 2023 Feb 7;28(4):1557–70. doi: 10.1038/s41380-023-01987-3 (PMC10208983; doi:10.1038/s41380-023-01987-3)
Supplement: Supplementary file 1 — Supplemental information [file 41380_2023_1987_MOESM1_ESM.docx]

**Supplementary materials and methods information**

**Generation of *WFS1* knockout hESCs with CRISPR/Cas9**

hESCs H1, H9 and HuES8 were grown on Matrigel-coated (Corning) plates and fed with mTeSR1 medium (Stemcell Technologies). One or two sgRNAs targeting one or two different loci on *WFS1* gene were cloned into P2U6 vectors. hESCs were transfected with the P2U6 plasmid carrying Cas9 and sgRNAs corresponding to the loci of *WFS1* gene. After electroporation, the hESCs were added 40 μg/ml puromycin for 2 days for selection. PCR was used to validate the knock-out efﬁciency. An extra nucleotide G was introduced into H1 and H9 at 70 bp on the 2^nd^ exon of *WFS1* separately, leading to frame-shift mutation and an early stop codon in the transcript; a 49 bp (from 19 bp to 67 bp) deletion was introduced on the 2^nd^ exon of *WFS1* in HuES8, resulting in a premature stop codon in the transcript. All cell lines were tested to be mycoplasma-free by PCR.

**Astrocyte differentiation**

NPCs were lifted off and gently dissociated by pipetting, and cultured in suspension to form neurospheres within N/B medium for 1 week on the shaker (95 r.p.m.). Next, astrocyte growth medium (AGM) (Lonza) was added to the neurospheres for 2 weeks and cells were maintained on the shaker (95 r.p.m.) to induce astrocytes differentiation. Medium was changed every other day. Spheres were plated on poly-L-ornithine/laminin-coated dishes, and astrocytes were migrated from spheres and maintained in AGM. Astrocytes were dissociated using Papain and DNase I (Worthington) for 30 min at 37 ℃ until analysis was performed.

**Electrophysiology**

For whole-cell patch-clamp recordings, the edge area of cerebral organoids was cut, transferred to a chamber which was perfused with ACSF. The chamber was plated on the stage of a microscope (Olympus) and oxygenated with 95% O_2_ and 5% CO_2_. The patch pipettes were 2.5–3.5 MΩ. The pipettes solutions contained (mM): 135 K-gluconate, 0.5 CaCl_2_, 2 MgCl_2_, 5 KCl, 5 EGTA, 5 HEPES, 5 D-glucose (pH adjusted to 7.3 with KOH). Spontaneous excitatory postsynaptic currents (sEPSCs) of cerebral organoids recordings were performed using an amplifier (Molecular Devices) under the voltage clamp. Cells were clamped at -70 mV. Signals were filtered at 2 kHz. All currents were sampled using a Digidata 1,550B interface. Traces were recorded in pClamp10.5 software (Molecular Devices).

**Generation of conditional *Wfs1* knockout mice**

C57BL/6J *Wfs1^flox/flox^* mice by employing CRISPR/Cas9 system were generated by Shanghai Model Organisms Center, Inc (Shanghai, China). A *Wfs1* donor vector containing flox sites flanking Exon 3 of *Wfs1* gene was created. Two sgRNA sites targeting Intron 2 and Intron 3 were *in vitro* transcribed. sgRNA target site for Intron 2 was 5'-TATACTGCGAGTGTGAGGAC TGG-3'. sgRNA site for Intron 3 was 5'-AGCTGAGACAGACCCTCCTT AGG-3'. The donor vector with two sgRNAs and Cas9 mRNA were microinjected into C57BL/6J fertilized eggs. F0 generation mice positive for homologous recombination were identified by long-PCR. The primers (P1-P2) used for genotyping the correct homology recombination were P1: 5'-TGCCCTCCAAAGCCACACTG -3' and P2: 5'-GGCCCTGCTACCTACTCCCATTTT-3' for the correct 5' homology arm recombination. The PCR products were further confirmed by sequencing. The genotype of F1 generation *Wfs1* flox heterozygous mice were identified by long-PCR. *Wfs1^flox/flox^*, *Nestin-Cre* mice were generated by crossing *Wfs1^flox/flox^* mice with *Nestin-Cre* mice. 3-4 months old female and male mice were used for all experiments unless specifically stated. Mice were maintained in a controlled environment (22 ± 1°C, 55 ± 5% humidity) with a 12 h:12 h light/dark cycle. All animal experiments performed in this study were compiled with ethical regulations and approved by the Biological Research Ethics Committee of Tongji University.

**Riluzole administration**

*Wfs1^flox/flox^*, *Nestin-cre* mice (CKO mice) and its control *Wfs1^flox/flox^* mice (WT mice) were randomly assigned to three groups: a. WT mice + vehicle; b. CKO mice + vehicle; c. CKO mice + Riluzole. Riluzole-treated CKO mice were given Riluzole hydrochloride (MedChemExpress) in drinking water at 50 mg/kg/day (previously tested in mice (1)) from the age of 3 weeks for 2 months. Vehicle-group mice consumed drinking water alone. All water bottles contained Riluzole were covered with foil to prevent light exposure, and fresh solution was changed every 48 h. All animals received food and water ad libitum.

**Behavioral studies**

**Forced swimming test**

Mice were individually placed into a transparent glass cylinder (15 cm diameter, 20 cm height) filled with tap water (22-23 ℃). The water depth was 13 cm to prevent mice from touching the cylinder bottom with tails or limbs. The test lasted for 6 min and were videotaped by a digital camera. The immobility time during the last 4 min was recorded. Immobility time was defined as when mice were motionless (no movement of all legs and the tail) or passively floating on the water (only with movements necessary for keeping balance).

**Novel object test**

The apparatus consisted of two objects placed at the ^1^/_4_ and ^3^/_4_ of one diagonal in a 25 cm x 25 cm x 25 cm box. On day 1, mice were allowed to acclimate in the four-squared box for 10 min. On day 2, mice were first allowed to familiar with the two same objects for 10 min in the session of acquisition. In the session of recognition, one of the two same objects was replaced by a novel one (different in color, shape and texture), and mice were allowed to explore for another 10 min. The activities of mice were recorded by a digital camera. The ability of recognition memory was analyzed as the ratio of the number of contacts with the novel object over the total number of contacts with the two objects.

**Water maze test**

The apparatus of water maze test was a circular pool (120 cm diameter, 47 cm height) filled with opaque water at a temperature of 22-23 °C. A transparent platform (9 cm diameter) was submerged 0.5 cm underneath the surface of water and placed in the same location for all trials. Different external cues were posted on the walls in the room. The software divided the pool into four quadrants. During the training sessions, mice were placed into the water at one of the four positions, which varied for each trial. Four trials per day were conducted with 1 hour interval for 7 consecutive days. Mice were allowed to swim for 60 s to find the platform in each trial. If the mouse failed to find the platform, it was guided to the platform and stayed on the platform for 30 s. Latency to find the platform was recorded for each trial, an indicator of spatial memory acquisition. On the probe trial day (Day 8), the platform was removed and mice were allowed to swim for 60 s. The latency to the original position of platform, the time spent in the quadrant of the platform and the times of crossing the platform area were analyzed.

**Lentivirus preparation and infection**

The SYN1::GFP and GFAP:: tdTomato vectors were obtained by cloning the Synapsin-1 promoter or the GFAP promoter into the vector. Lentivirus destination plasmid was co-transfected with the mixture of packaging plasmids (PSPAX2 and PMD2.G) into HEK293FT cells using calcium phosphate cell transfection protocol (system containing 250 μl ddH_2_O of 7.5 μg PSPAX2, 3 μg PMD2.G, 10 μg destination plasmid and 31 μl 2.5 M CaCl_2_; 400 μl of 2× HEPES (100 ml ddH_2_O containing 0.8 g NaCl, 0.022 g Na_2_HPO_4_ and 1.2 g HEPES, pH=7.05). The medium was replaced with DMEM containing 10% FBS at 12 h after transfection. After 60 h, viral particles in the supernatant were collected, filtered through 0.45 μm filters (Millipore) and concentrated by Ultracentrifuge (Beckman Coulter, Optima XPN-100) (28,000 *g* for 2 h at 4 °C). Lentivirus was stored in aliquots at −80 °C until use. NPCs were infected with lentivirus SYN1::GFP or GFAP:: tdTomato for 24 h. Infected NPCs were further differentiated to neurons or astrocytes as needed.

**Co-culture experiments**

Astrocytes and lentivirus SYN1::GFP infected neurons were used for co-culture experiments. Astrocytes were plated at 3×10^5^ per well in a 12-well plate containing poly-L-ornithine/laminin-coated coverslips, after 2 days, 2×10^5^ neurons were plated on the top of astrocytes and kept for 3 days in AGM before fixation.

**Library construction and NGS sequencing**

Neurons expressing SYN1::GFP were digested using Neurosphere Dissociation Kit (Miltenyi Biotec) according to the manufacturer’s instructions and astrocytes expressing GFAP::tdTomato were digested using Papain and DNase I (Worthington). SYN1-GFP^+^ or GFAP-tdTomato^+^ single cells were sorted using Moflo Astrios (Beckman Coulter) and collected in RNase-free 96-well-plate. An optimized Smart-seq2 protocol was used to construct single-cell RNA-seq libraries while sample pooling and molecular counting were using oligo-d (T) primers comprising 16-bp cell barcode sequence and 9-bp molecular barcode sequence. For compatible with Illumina sequence platform, part of the ISPCR sequence was replaced by read2 sequence in the oligo-d(T) primer. Sorted neuron and astrocytes were denatured in lysis buffer on 72 °C for 3 min, and put on ice for annealing immediately, then reverse transcribed with template switching oligos and pre-amplified with both ISPCR and read2 primers. cDNA libraries from different cells with different barcodes were pooled together and purified with 0.8× AMPure XP beads. Purified cDNA was fragmented with homemade Tn5 enzyme, and final amplification using Tn5PCR-7 primers and Tn5PCR-5 primers (Nextera). Amplificated cDNA libraries were purified twice with AMPure XP beads. The final libraries were sequenced on Illumina HiSeq X10 platform.

**Single-cell RNA-seq data processing**

Reads were stored in paired-end fastq format. Cell barcode and UMI information in R2 was extracted and added to the name of corresponding reads in R1 which containing molecular sequence. In this way, we integrated the information in R1 and R2 and generated single-end fastq files. Then, using Trim Galore (http://www.bioinformatics.babraham.ac.uk/projects/trim_galore/), sequences of adaptors were trimmed and reads shorter than 30 bp were discarded.

Subsequently, we employed FastQC (<https://www.bioinformatics.babraham.ac.uk/projects/fastqc/>) to check the quality of retained reads and aligned them on hg19 reference genome using STAR (2). The parameters of STAR software were --AlignEndsType EndToEnd --out FileterMismatchNoverReadLmax 0.04 --outSAMattrIHstart 0 --outSAMmultNmax 1 --outFilterMultimapNmax 1. GTF file of hg19 reference genome was derived from the RefSeq (3) gene annotation file downloaded from UCSC genome browser database (http://genome.ucsc.edu/cgi-bin/hgTables?db=hg19). The SAM format files generated by the alignment step were used for demultiplexing and reads were sorted into individual cells by Cutadapt (4) with parameters --overlap 16 –no-indels --match-read-wildcards and reads assigned to more than one cells were discarded. In addition, we removed PCR duplicates by UMI-tools (5) and quantified gene expression levels according to the above mentioned GTF file using featureCounts (6). After all these steps, the UMI count matrix was generated. Cells with fewer than 200 genes with ≥ 2 UMI count were considered as low-quality and were filtered out. 746 of the 1,152 collected cells passed this quality control step, with a median total count of 5,128 for the 539 neurons and 11,377 for the 207 astrocytes. Then, the scRNA-seq profiles of these two different types of cells were processed and analyzed separately.

**Single-cell RNA-seq data analysis**

Genes with ≥ 2 UMIs in at least 3 cells were retained, resulting a UMI count matrix of 11,785 genes by 539 cells for neurons and a matrix of 10,620 genes by 207 cells for astrocytes. Then, these two raw count matrices were normalized by cell-specific size factors, which were calculated as the total count of individual cells divided by the mean of cells’ total count.

**Clustering neurons**

For neurons, data from two batches were integrated and scaled using the function rescaleBatches in R package batchelor (7) and then processed by Seurat 3.2.3 (8) for dimensional reduction. In detail, principal components analysis (PCA) was carried out with the top 2,000 most variable genes, and the top 10 PCs were used for uniform manifold approximation and projection (UMAP) (9) and cell clustering. Two clusters of neurons were detected and differential gene expression analysis was carried out on the raw count data between cells of these two clusters. In detail, the R package edgeR (10) was used in combined with zingeR (https://github.com/statOmics/zinbwaveZinger) to incorporate an estimate of the dropout rate per cell (edgeR-zingeR) with the batch as a covariate (11). Based on the results of differential expression analysis, gene set enrichment analysis (GSEA) (12) was performed on the pre-ranked genes list. Specifically, genes were ranked according to their z-statistics derived from p-values multiplied by the sign of the log_2_FC generated by edgeR-zingeR and then significantly enriched GO terms were detected as those overrepresented at the extremes (top or bottom) of the ranked list of genes. Specifically, GO terms with FDR≤0.05 were identified as significantly enriched.

**Constructing trajectory for neurons**

Trajectory of neurons was constructed using Monocle 2.18.0 (13-15) through the following three steps. 1) Genes that define a cell’s progress were chosen by the differential test between WT and *WFS1^-/-^* using differentialGeneTest function. 2) Data dimensionality was reduced using reduceDimension function with the method “DDRTree”. 3) Single neurons were ordered along the trajectory. Then, the function differentialGeneTest was called again to find genes whose expression that changed as a function of the pseudo-time and 1931 genes with qvalue≤0.05 were detected to have significantly correlated expression with pseudo-time.

**Analyzing data of astrocytes**

For the analysis of data of astrocytes, because all astrocytes were from the same batch, the same steps and tools were used as those for the data of neurons, except for those related to batch effect removal. Dimensional reduction was performed directly using normalized data and differential analysis between astrocytes of *WFS1^-/-^* and WT using edgeR-zingeR without taking the batch as a covariate. Differentially expressed genes were those with FDR ≤ 0.05 and |log_2_FC| > 0.58.

**Code availability**

Raw scRNA-seq data (fastq files) and processed expression matrix in this study have been submitted to NCBI’s Gene Expression Omnibus (GEO) and are accessible through accession numbers: GSE185052.

**Quantitative real-time PCR**

Total RNA was isolated using TRNzol (Tiangen) and reverse-transcribed into cDNA by FastQuant RT Kit (Tiangen). Quantitative real-time PCR was performed using SuperReal PreMix Plus (SYBR Green) (Tiangen) on Lightcycler 96 (Roche). The amplification efficiency for each primer and the cycle threshold (Ct) were determined automatically by Lightcycler software (Roche). The fold-change was calculated by the comparative CT (2^−ΔΔCT^) method against GAPDH. Primers used for this study were listed as below (forward primer sequence, FW; reverse primer sequence, RE): *GFAP* FW: AGTCCCTGGAGAGGCAGATGCGCGAGC; *GFAP* RE: ATGTTCCTCTTGAGGTGGCCTTCTGAC. *EAAT2* FW: TAGCCGCCATCTTTATAGCCC; *EAAT2* RE: CGGCTGTCAGAATGAGGAGC. *PERK* FW: GTCCGGAACCAGACGATGAG; *PERK* RE: GGCTGGATGACACCAAGGAA. *ATF6* FW: CCGCAGAAGGGGAGACACA; *ATF6* RE: TCGGAGGTAAGGAGGAACTGACG. *IRE1* FW: CACAGTGACGCTTCCTGAAAC; *IRE1* RE: GCCATCATTAGGATCTGGGAGA.

**Immunofluorescence staining**

For adherent cells, cells were fixed in 4% paraformaldehyde (PFA) for 15 min at RT and washed with PBS three times. Cells were then permeabilized with 0.1% triton X-100 in PBS at RT for 15 min and washed with PBS three times. Cells were blocked in 5% normal donkey serum (Jackson) in PBS at RT for 2 h. Primary antibody incubation was performed at 4 °C overnight. The next day, cells were washed with PBS three times, then secondary antibodies were performed in the dark at RT for 1 h. For cerebral organoids, organoids were fixed in 4% PFA at 4 °C overnight, and then dehydrated with 30% sucrose in PBS at 4 °C overnight. Organoids were then embedded in OCT (SAKURA) for cryosectioning at 16 μm with Leica CM1950. Sections were blocked and permeabilized in 5% normal donkey serum and 0.1% Triton X-100 in PBS (PBST) at RT for 1 h, followed by incubation with primary antibody at 4 °C overnight. After being washed three times for 10 min with PBST, sections were incubated with secondary antibodies at RT for 1 h. Nuclei were stained using DAPI. Primary antibodies used in this study included: goat SOX2 (1:1000, R&D, AF2018), chicken MAP2 (1:500, abcam, ab5392), rabbit Ki67 (1:400, thermo, MA5-14520), rabbit cleaved caspase-3 (1:200, Cell Signaling Techonology, 9664S), mouse Nanog (1:100, Cell Signaling Techonology, 1E6C4), rabbit Oct4 (1:100, abcam, ab19857), rabbit GFAP (1:500, Dako, Z0334), mouse GFAP (1:400, Sigma, G3893), mouse Synapsin 1 (1:500, synaptic systems, 106001), rabbit PSD95 (1:500, abcam, ab18258), rabbit P50 (1:500, abcam, ab32360), rabbit P65 (1:100, abways, CY5034),. Images were captured using a fluorescence microscope (Zeiss Axio Imager M2) and confocal microscope (Zeiss LSM 880 and Leica SP8). Images were analyzed using ImageJ software.

**Quantification of immunofluorescence image**

To determine the percentage of specific markers, sections of cerebral organoids or coverslips containing differentiated cells were stained and imaged for quantification. The number of cells expressing different markers was manually counted using “Point tool” in ImageJ or automatically counted by setting the suitable threshold for individual channels. SYN1 puncta per field were manually counted using ImageJ, and only puncta colocalized with MAP2-positive processes were scored. The number of MAP2^+^ cells were measured for normalization. SYN1/PSD95 colocalization puncta per field were manually counted using ImageJ, and only PSD95 puncta within 2 μm from SYN1 signals were quantified in MAP2-positive processes. The number of SYN1 puncta were measured for normalization. Morphology analysis of neurons was traced using “NeuronJ” plugin in ImageJ, and total dendritic length was calculated by summing total length of all dendrites per neuron. The number of dendrite branches was calculated by adding up total number of dendritic segments per neuron.

**Glutamate assay**

3×10^5^ per well astrocytes were plated in a 12-well plate. Medium was collected from astrocytes at intervals of 48 and 96 h after fresh medium was changed. Glutamate assay was performed using glutamate assay kit according to the manufacturer’s instructions (Sigma) and measured at 450 nm using microplate reader. The assay normalization was based on staining quantification of GFAP-positive astrocytes, performed after the last collection of medium. Quantification analysis was performed using ImageJ.

**Calcium imaging**

Cells were washed with artificial cerebrospinal fluid (ACSF) containing 126 mM NaCl, 2.5 mM KCl, 26 mM NaHCO_3_, 2 mM CaCl_2_, 2 mM MgCl_2_, 1.25 mM NaH_2_PO_4_, and 10 mM glucose. Neurons only were loaded with 5 μM Fluo-4/AM (Invitrogen) for 1 h at 37 °C in ACSF. Neurons labeled with lentivirus SYN1::GFP in co-culture were loaded with 5 μM Rhod-4/AM (AAT Bioquest) for 1 h at 37 °C in ACSF. Time-lapse Ca^2+^ measurements were carried out in ACSF using a confocal microscope (Leica SP8) with 40 × objectives. Frames (1024 × 1024 pixels) taken every 2 sec for 200 frames. Calcium activity analysis was performed using ImageJ (Fiji). Regions of interest (ROIs) were manually selected. Fluorescence intensity (F) was the mean grey values in ImageJ. Changes in fluorescence was calculated as follows: ΔF/F = (F- F_basal_))/F_background_ where F_basal_ was the lowest fluorescence value per cell across imaging while F_background_ was the averaged mean fluorescence of the background across all frames. The amplitude of Ca^2+^ signals was calculated as the mean fluorescence intensity during 200 frames. A ΔF > 0.2 was defined as a spike. An signaling cell had one or more Ca^2+^ spikes.

**Electron microscopy**

Cerebral organoids or neurons were fixed in fresh TEM fixative. Then cells were washed three times (15 min each) with 0.1 M PB (pH 7.4), and cells were fixed with 1% OsO4 in 0.1 M PB (pH 7.4) for 2 h at room temperature. Next, cells were rinsed in 0.1 M PB (pH 7.4) for 3 times (15 min each), and were dehydrated at room temperature as followed: 30%-50%-70%-80%-95%-100%-100% ethanol for 20 min each; acetone for 15 min twice. Then cells were penetrated and embedded with acetone and EMBed 812. Finally, cells were put into the embedding models with pure EMBed 812, and then kept at 37°C overnight and were polymerized at 65°C for 48 h. 60-80 nm sections were cut by ultramicrotome and stained with 2% uranium acetate saturated alcohol solution for 8 min and 2.6% lead citrate for 8 min. Images were captured using a transmission electron microscope (JEM-1230).

**Flow cytometry analysis**

Cells were washed and fixed with 1.6% PFA for 20 min at 37°C, followed by two washes in FACS buffer (0.5% BSA in PBS). Cells were washed twice with Intracellular Staining Perm Wash Buffer (Perm Buffer) (Biolegend) and then incubated with primary antibodies diluted in FACS buffer for 30 min at RT followed by two washes in Perm Buffer. Cells were then incubated with the appropriate secondary antibodies for 30 min at RT followed by two washes in Perm Buffer. Cells were then resuspended in FACS buffer before analysis on CytoFLEX LX (Beckman Coulter). Data were analyzed with FlowJo V10.0.7 software.

**Luciferase assay**

The luciferase reporter vector pGL3 NF-κB contained five copies of an NF-κB response element that drives transcription of the luciferase reporter gene. A total of 2.5×10^5^ astrocytes per well were seeded in 24-well plate and were transfected with 1 μg total DNA using lipo3000 (Invirtogen) according to the manufacturer’s protocol. 500 ng pGL3 NF-κB or the empty vector pGL3 was transfected into astrocytes with 500 ng Renilla luciferase vector. After overnight transfection, cells were switched to the fresh astrocyte growth medium, and proceeded with PDTC treatment (100 μM for 40 min), Thapsigargin treatment (0.125 μM for 24 h) or Riluzole treatment (5 μM for 48 h). Luciferase activity was measured with the Dual Luciferase Reporter Gene Assay Kit (Beyotime) according to the manufacturer’s instructions. Relative firefly luciferase activity was calculated by normalizing to Renilla luciferase activity.

**Magnetic cell sorting**

Differentiated neurons in the 3-week neuron cultures was quantified by magnetic-activated cell sorting for CD44 and CD184 double-negative (CD44^-^CD184^-^) cells using Neuron Magnetic Sorting Protocol (Miltenyi Biotec) according to the manufacturer’s instructions. Briefly, dissociated neurons were stained with 30 μl CD184PE and 30 μl CD44PE (Miltenyi Biotec) on ice, in the dark for 15 min. Then cells were incubated with 20 μl Anti-PE Microbeads (Miltenyi Biotec) for 15 min at 4 ℃. For magnetic separation, MACS Column (Miltenyi Biotec) was placed into the magnetic field of the MidiMACS Separator (Miltenyi Biotec), and cell suspension was applied onto the prepared Column. Collected flow-through containing unlabeled cells were CD44^-^CD184^-^ cells. The percentage of CD44^-^CD184^-^ cells was calculated by the cell number of the unlabeled cells (CD44^-^CD184^-^ cells) divided by the total cell number of neurons before sorting.

**Supplementary figure legends**

**Figure S1. Generation and characterization of *WFS1*-deficient hESCs**

(A) Schematic representation of *WFS1* showing the position and deletion region of *WFS1^-/-^* hESCs. (B) Representative bright field images of HuES8 and H1 hESCs. Scale bar, 50 μm. (C) Immunostaining for the pluripotent markers Nanog (green) and OCT4 (red) in HuES8 and H1 hESCs. Scale bar, 40 μm. (D) H&E staining of teratoma tissue derived from HuES8 and H1 hESCs to test for pluripotency. Scale bar, 100 μm. (E) Cell proliferation curve of HuES8 and H1 hESCs, n = 3 independent experiments. (F) Analyses summary of three independent hESCs used in this study. Data are presented as mean ± SD. *p* values calculated by unpaired two-tailed Student’s *t* test were **p* < 0.05, ***p* < 0.01, and ****p* < 0.001.

**Figure S2. *WFS1* deficiency reduces organoid size and astrocytes but increases neuronal apoptosis in cerebral organoids derived from H9 hESCs**

(A) Representative bright-field images of WT and *WFS1^-/-^* cerebral organoids at Day 30, Day 60, Day 100, Day 140, Day 180 and Day 200. Scale bar, 1 mm. (B) Quantification of the individual area (mm^2^) of WT and *WFS1^-/-^* cerebral organoids, n ≥ 10 individual organoids. (C) Immunostaining for SOX2 (green), Ki67 (red) and DAPI (blue) at Day 30. Scale bar, 50 μm. (D) Quantification of the percentage of Ki67^+^ cells among the total number of SOX2^+^ NPCs in WT and *WFS1^-/-^* cerebral organoids at Day 30, n = 3 individual organoids. (E) Immunostaining for SOX2 (green), CAS3 (red) and DAPI (blue) at Day 30. Scale bar, 50 μm. (F) Quantification of the percentage of CAS3^+^ cells among the total number of SOX2^+^ NPCs in WT and *WFS1^-/-^* cerebral organoids at Day 30, n = 3 individual organoids. (G) Immunostaining for GFAP (green) and DAPI (blue) in WT and *WFS1^-/-^* cerebral organoids at Day 60. Scale bar, 50 μm. (H) Quantification of the percentage of GFAP^+^ astrocytes among the total number of DAPI^+^ cells in WT and *WFS1^-/-^* cerebral organoids at Day 60, n = 3 individual organoids. (I) Immunostaining for MAP2 (magenta) and DAPI (blue) in WT and *WFS1^-/-^* cerebral organoids at Day 60. Scale bar, 50 μm. (J) Quantification of the percentage of MAP2^+^ neurons among the total number of DAPI^+^ cells in WT and *WFS1^-/-^* cerebral organoids at Day 60, n = 3 individual organoids. (K) Immunostaining for GFAP (green), MAP2 (red) and DAPI (blue) in WT and *WFS1^-/-^* cerebral organoids at Day 210. Scale bar, 50 μm. (L) Quantification of the percentage of GFAP^+^ astrocytes among the total number of DAPI^+^ cells in WT and *WFS1^-/-^* cerebral organoids at Day 210, n = 3 individual organoids. (M) Quantification of the percentage of MAP2^+^ neurons among the total number of DAPI^+^ cells in WT and *WFS1^-/-^* cerebral organoids at Day 210, n = 3 individual organoids. Data are presented as mean ± SD. *p* values calculated by unpaired two-tailed Student’s *t* test were **p* < 0.05, ***p* < 0.01, and ****p* < 0.001.

**Figure S3. *WFS1* deficiency reduces rosette numbers and layer thickness, and increases undifferentiated NPCs population**

(A) Representative immunostaining for SOX2 (green), MAP2 (red) and DAPI (blue) in WT and *WFS1^-/-^* cerebral organoids at Day 50. Scale bar, 100 μm. (B) Representative immunostaining for SOX2 (green), MAP2 (red) and DAPI (blue) in the whole WT and *WFS1^-/-^* cerebral organoids at Day 50. Scale bar, 400 μm. (C and D) Quantification of the layer thickness of VZ-like zones and the number of rosettes in WT and *WFS1^-/-^* cerebral organoids at Day 50, n ≥ 3 individual organoids. (E) Immunostaining for GFAP (green), MAP2 (red), SOX2 (magenta) and DAPI (blue) in WT and *WFS1^-/-^* cerebral organoids at Day 90 and Day 170. Scale bar, 25 μm. (F) Quantification of the percentage of SOX2^+^ cells among the total number of DAPI^+^ cells in WT and *WFS1^-/-^* cerebral organoids at Day 90 and Day 170, n ≥ 3 individual organoids. (G) Quantification of the percentage of SOX2^+^GFAP^-^MAP2^-^ cells among the total number of DAPI^+^ cells in WT and *WFS1^-/-^* cerebral organoids at Day 90 and Day 170, n ≥ 3 individual organoids. (H) The overall view of the whole cerebral organoid at Day 60, Day 90 and Day 170 by immunostaining of GFAP (green), MAP2 (red) and DAPI (blue). Scale bar, 1 mm. Data are presented as mean ± SD. *p* values calculated by unpaired two-tailed Student’s *t* test were **p* < 0.05, ***p* < 0.01, and ****p* < 0.001. Two-way ANOVA was used for analysis of the time effect on the percentage of SOX2^+^ cells and SOX2^+^GFAP^-^MAP2^-^ cells, **p* < 0.05, ***p* < 0.01, and ****p* < 0.001 for the comparison of WT and *WFS1^-/-^* organoids, #*p* < 0.05, ##*p* < 0.01, and ###*p* < 0.001 for the comparison of the effect of time.

**Figure S4. Cell-autonomous detrimental effects of *WFS1* deficiency on neurons**

(A) Table summary of the number of WT and *WFS1^-/-^* neurons in each batch, as well as the situation of quality control of cells in each 96-well plate. (B) Scatter plot of expression of several psychiatric disorders associated genes over pseudo-time. Each dot represents a single neuron. Cells are color coded for the corresponding conditions (WT or *WFS1^-/-^*). (C) Immunostaining for CAS3 (green), MAP2 (red) and DAPI (blue) in WT and *WFS1^-/-^* neurons. Scale bar, 50 μm. (D) Quantification of the percentage of CAS3^+^ cells among the total number of MAP2^+^ neurons, n = 3 independent experiments. (E and F) Representative flow cytometry analysis and quantification of the percentage of SOX2^+^ cells in WT and *WFS1^-/-^* neurons, n = 5 independent experiments. (G) Quantification of the percentage of CD44^-^CD184^-^ cells in WT and *WFS1^-/-^* neurons by magnetic-activated cell sorting, n = 7 independent experiments. Data are presented as mean ± SD. *p* values calculated by unpaired two-tailed Student’s *t* test were **p* < 0.05, ***p* < 0.01, and ****p* < 0.001.

**Figure S5. *WFS1* deficiency impairs neurite outgrowth *via* the elevation of cytosolic Ca^2+^**

(A) Representative single cell traces of intracellular spontaneous calcium activity of WT neurons treated with 0.125 μM Thapsigargin or vehicle. (B-D) Intracellular spontaneous calcium activity analysis shown as calcium spike frequency (B), mean fluorescence intensity (C) and the percentage of signaling neurons (D) in neurons, n = 6 fields from 3 independent experiments. (E) Representative images of WT neurons treated with 0.125 μM Thapsigargin or vehicle stained with MAP2 (red). Scale bar, 20 μm. (F-G) Quantification of the total dendritic length (μm) and the number of dendrite branches, n = 3 independent experiments. (H) Representative single cell traces of intracellular spontaneous calcium activity of *WFS1^-/-^* neurons treated with 8 μM Dantrolene or vehicle. (I-K) Intracellular spontaneous calcium activity analysis shown as calcium spike frequency (I), mean fluorescence intensity (J) and the percentage of signaling neurons (K) in neurons, n = 6 fields from 3 independent experiments. (L) Representative images of *WFS1^-/-^* neurons treated with 8 μM Dantrolene or vehicle stained with MAP2 (red). Scale bar, 25 μm. (M-N) Quantification of the total dendritic length (μm) and the number of dendrite branches, n = 3 independent experiments. Data are presented as mean ± SD. *p* values calculated by unpaired two-tailed Student’s *t* test were **p* < 0.05, ***p* < 0.01, and ****p* < 0.001.

**Figure S6. Non-cell-autonomous detrimental effects of *WFS1*-deficient astrocytes**

(A) Table summary of the number of WT and *WFS1^-/-^* astrocytes, and the situation of quality control of cells in each 96-well plate. (B) Representative images of WT neurons co-cultured with WT/*WFS1^-/-^* astrocytes stained with MAP2 (green), GFAP (red) and DAPI (blue). Scale bar, 20 μm. (C) Representative images of co-culture of WT neurons and WT/*WFS1^-/-^* astrocytes, neurons labeled with lentivirus SYN1::GFP were stained with MAP2 (magenta). Scale bar, 50 μm. (D) Quantification of the percentage of MAP2^+^ cells among the total number of SYN1-GFP^+^ neurons, results were normalized to WT astrocytes and WT neurons group, n = 4 independent experiments. (E) Immunostaining for P50 or P65 (green), GFAP (red) and DAPI (blue) in WT and *WFS1^-/-^* astrocytes. Scale bar, 50 μm. (F and G) Quantification of the intensity of P50 and P65 in nuclei area of WT and *WFS1^-/-^* astrocytes, n = 3 independent experiments. (H) NF-κB luciferase activity of *WFS1^-/-^* astrocytes treated with 100 μM PDTC or vehicle, n = 4 independent experiments. (I) Quantitative real-time PCR analysis of *PERK*, *ATF6* and *IRE1* in WT and *WFS1^-/-^* astrocytes, n = 5 independent experiments. (J) NF-κB luciferase activity of WT astrocytes treated with 0.125 μM thapsigargin or vehicle, n = 6 independent experiments. Data are presented as mean ± SD. *p* values calculated by unpaired two-tailed Student’s *t* test were **p* < 0.05, ***p* < 0.01, and ****p* < 0.001.

**Figure S7. Glutamate toxicity impairs neurite outgrowth with elevated cytosolic Ca^2+^ and the effect of Riluzole on neuronal loss in co-culture**

(A) Representative images of one typical WT neuron treated with 100 μM glutamate or vehicle, cells were stained with MAP2 (red) and DAPI (blue). Scale bar, 20 μm. (B and C) Quantification of the total dendritic length (μm) and the number of dendrite branches, n = 3 independent experiments. (D) Representative images of WT neurons treated with 100 μM glutamate or vehicle, cells were stained with MAP2 (red) and DAPI (blue). Scale bar, 50 μm. (E) Quantification of the percentage of MAP2^+^ cells among the total number of DAPI^+^ cells, n = 3 independent experiments. (F) Representative single cell traces of intracellular spontaneous calcium activity of WT neurons treated with 100 μM glutamate or vehicle. (G-I) Intracellular spontaneous calcium activity analysis shown as calcium spike frequency (G), mean fluorescence intensity (H) and the percentage of signaling neurons (I) in neurons, n = 6 fields from 3 independent experiments. (J) Representative images of co-culture of WT/*WFS1^-/-^* astrocytes and WT neurons treated with 5 μM riluzole and vehicle, neurons labeled with lentivirus SYN1::GFP were stained with MAP2 (magenta). Scale bar, 50 μm. (K) Quantification of the percentage of MAP2^+^ cells among the total number of SYN1-GFP^+^ neurons, results were normalized to WT astrocytes and WT neurons group, n = 3 independent experiments. Data are presented as mean ± SD. *p* values calculated by unpaired two-tailed Student’s *t* test were **p* < 0.05, ***p* < 0.01, and ****p* < 0.001.

**References**

1. Ishiyama T, Okada R, Nishibe H, Mitsumoto H, Nakayama C. Riluzole slows the progression of neuromuscular dysfunction in the wobbler mouse motor neuron disease. Brain Res. 2004;1019(1-2):226-36.

2. Dobin A, Davis CA, Schlesinger F, Drenkow J, Zaleski C, Jha S, et al. STAR: ultrafast universal RNA-seq aligner. Bioinformatics. 2013;29(1):15-21.

3. Pruitt KD, Tatusova T, Maglott DR. NCBI Reference Sequence (RefSeq): a curated non-redundant sequence database of genomes, transcripts and proteins. Nucleic Acids Res. 2005;33(Database issue):D501-4.

4. Marcel.M. Cutadapt Removes Adapter Sequences From High-Throughput Sequencing Reads. EMBnet journal. 2011;1(1):10-2.

5. Smith T, Heger A, Sudbery I. UMI-tools: modeling sequencing errors in Unique Molecular Identifiers to improve quantification accuracy. Genome Res. 2017;27(3):491-9.

6. Liao Y, Smyth GK, Shi W. featureCounts: an efficient general purpose program for assigning sequence reads to genomic features. Bioinformatics. 2014;30(7):923-30.

7. Haghverdi L, Lun ATL, Morgan MD, Marioni JC. Batch effects in single-cell RNA-sequencing data are corrected by matching mutual nearest neighbors. Nat Biotechnol. 2018;36(5):421-7.

8. Stuart T, Butler A, Hoffman P, Hafemeister C, Papalexi E, Mauck WM, 3rd, et al. Comprehensive Integration of Single-Cell Data. Cell. 2019;177(7):1888-902 e21.

9. Becht E, McInnes L, Healy J, Dutertre CA, Kwok IWH, Ng LG, et al. Dimensionality reduction for visualizing single-cell data using UMAP. Nat Biotechnol. 2018.

10. Robinson MD, McCarthy DJ, Smyth GK. edgeR: a Bioconductor package for differential expression analysis of digital gene expression data. Bioinformatics. 2010;26(1):139-40.

11. Vieth B, Parekh S, Ziegenhain C, Enard W, Hellmann I. A systematic evaluation of single cell RNA-seq analysis pipelines. Nat Commun. 2019;10(1):4667.

12. Subramanian A, Tamayo P, Mootha VK, Mukherjee S, Ebert BL, Gillette MA, et al. Gene set enrichment analysis: a knowledge-based approach for interpreting genome-wide expression profiles. Proc Natl Acad Sci U S A. 2005;102(43):15545-50.

13. Trapnell C, Cacchiarelli D, Grimsby J, Pokharel P, Li S, Morse M, et al. The dynamics and regulators of cell fate decisions are revealed by pseudotemporal ordering of single cells. Nat Biotechnol. 2014;32(4):381-6.

14. Qiu X, Hill A, Packer J, Lin D, Ma YA, Trapnell C. Single-cell mRNA quantification and differential analysis with Census. Nat Methods. 2017;14(3):309-15.

15. Qiu X, Mao Q, Tang Y, Wang L, Chawla R, Pliner HA, et al. Reversed graph embedding resolves complex single-cell trajectories. Nat Methods. 2017;14(10):979-82.
