# Supplementary figures and images for: Modeling disrupted synapse formation in wolfram syndrome using hESCs-derived neural cells and cerebral organoids identifies Riluzole as a therapeutic molecule
Source: Mol Psychiatry. 2023 Feb 7;28(4):1557–70. doi: 10.1038/s41380-023-01987-3 (PMC10208983; doi:10.1038/s41380-023-01987-3)

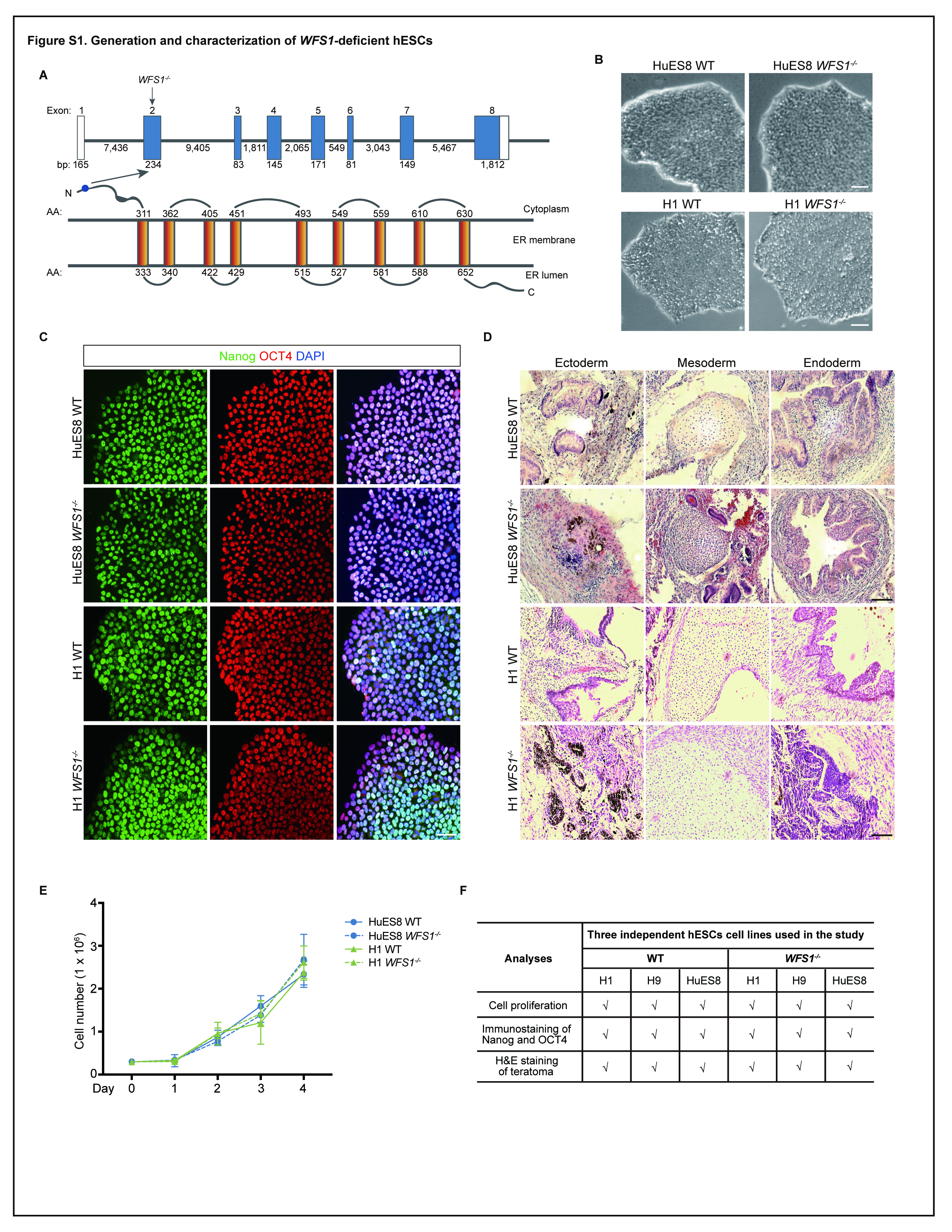

Supplement: Supplementary file 2 — Supplementary figure 1 [file 41380_2023_1987_MOESM2_ESM.tif]

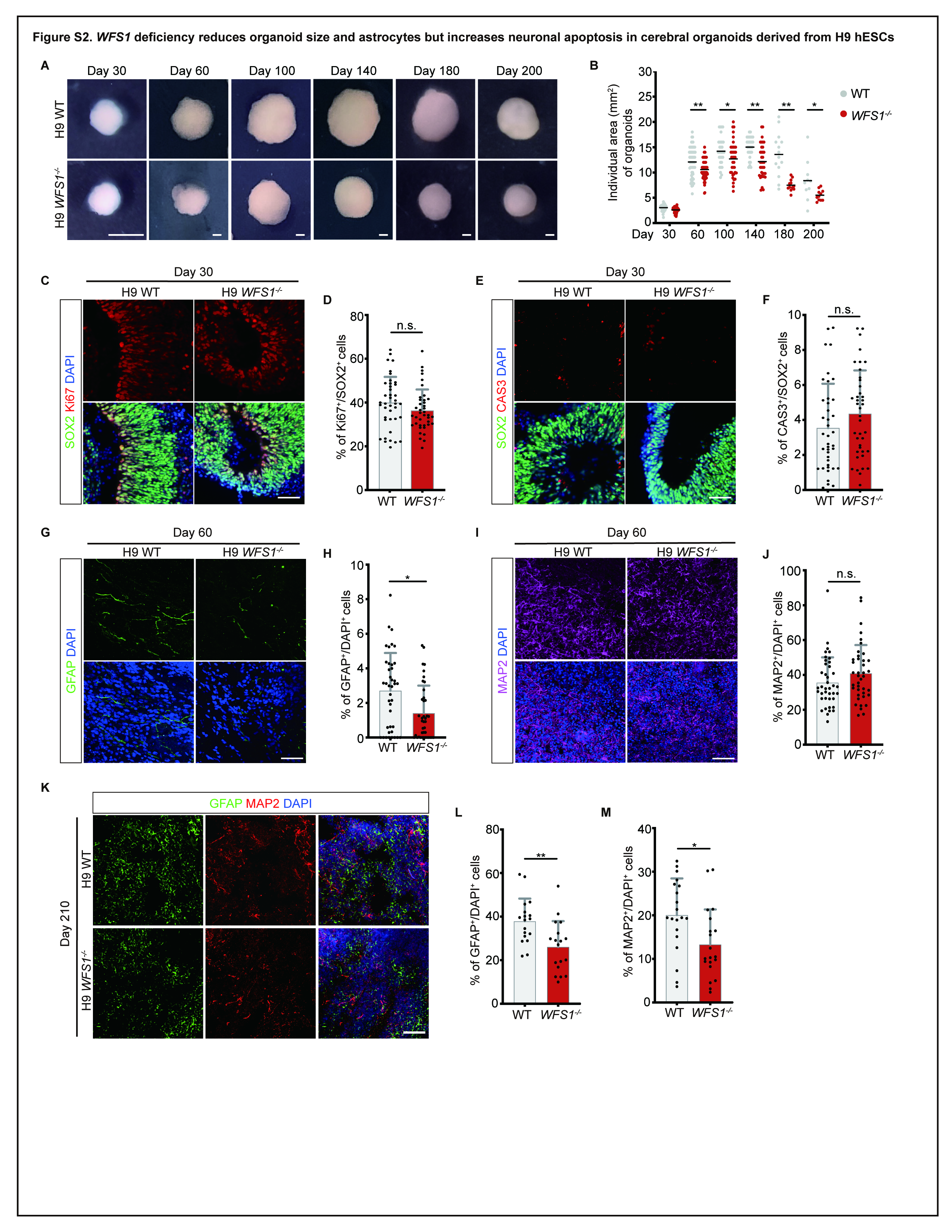

Supplement: Supplementary file 3 — Supplementary figure 2 [file 41380_2023_1987_MOESM3_ESM.tif]

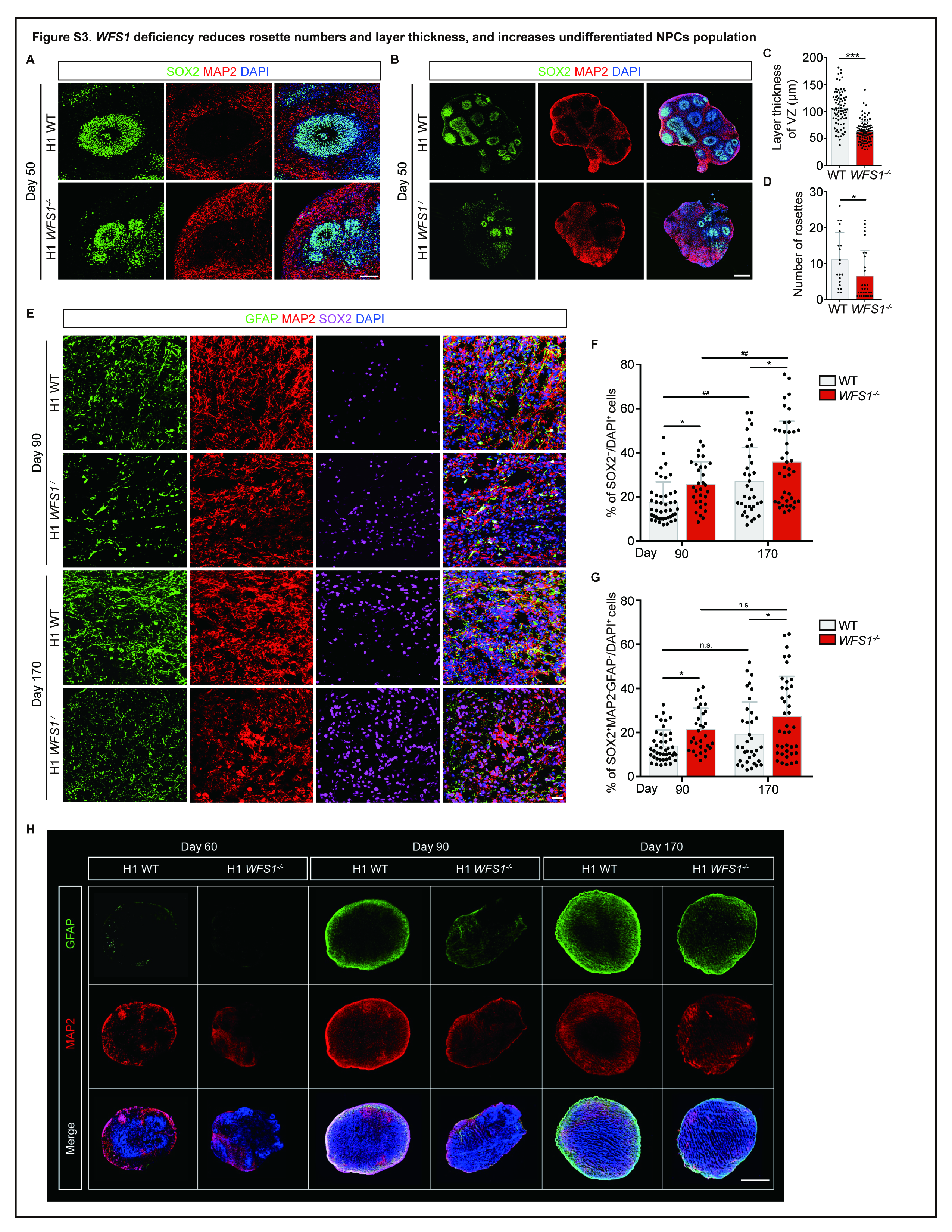

Supplement: Supplementary file 4 — Supplementary figure 3 [file 41380_2023_1987_MOESM4_ESM.tif]

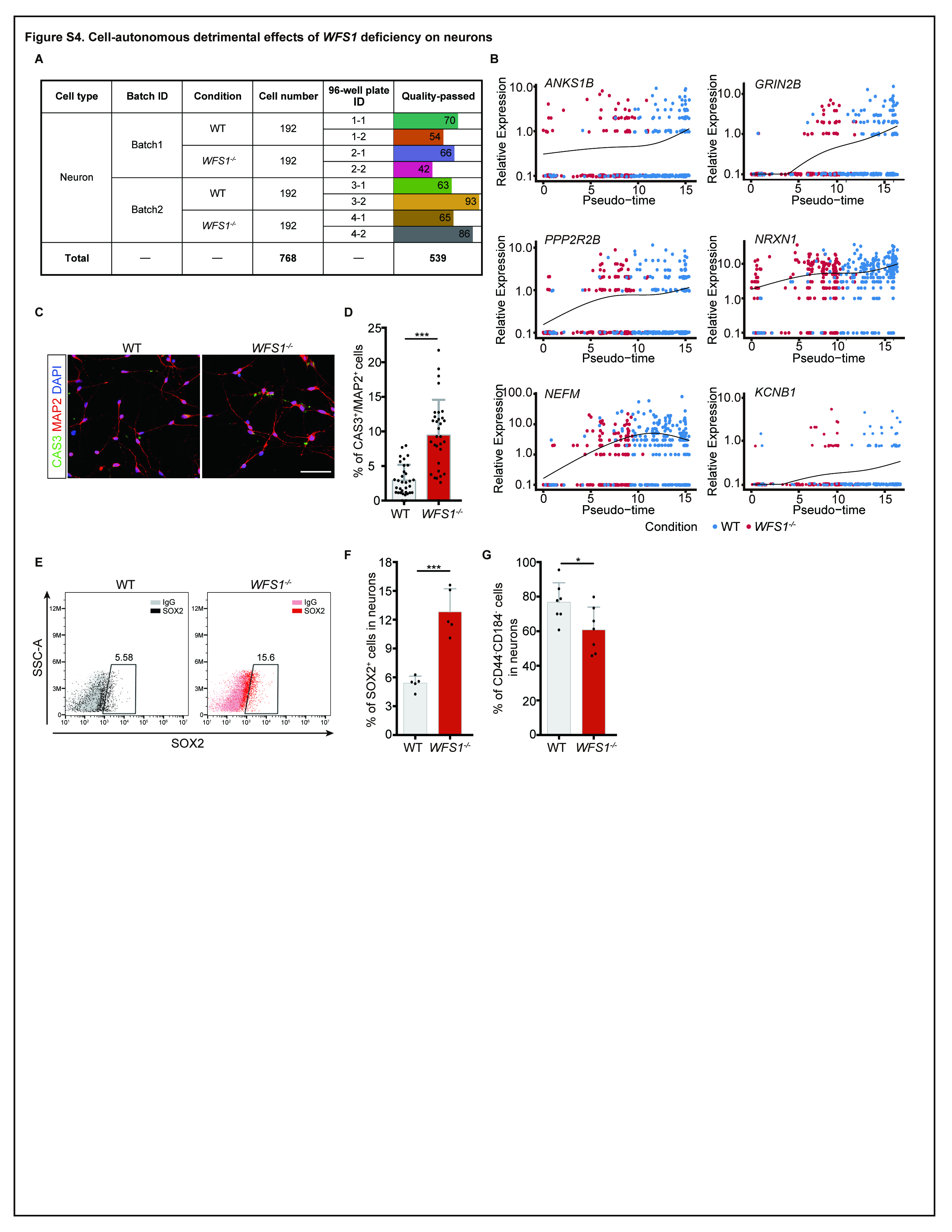

Supplement: Supplementary file 5 — Supplementary figure 4 [file 41380_2023_1987_MOESM5_ESM.tif]

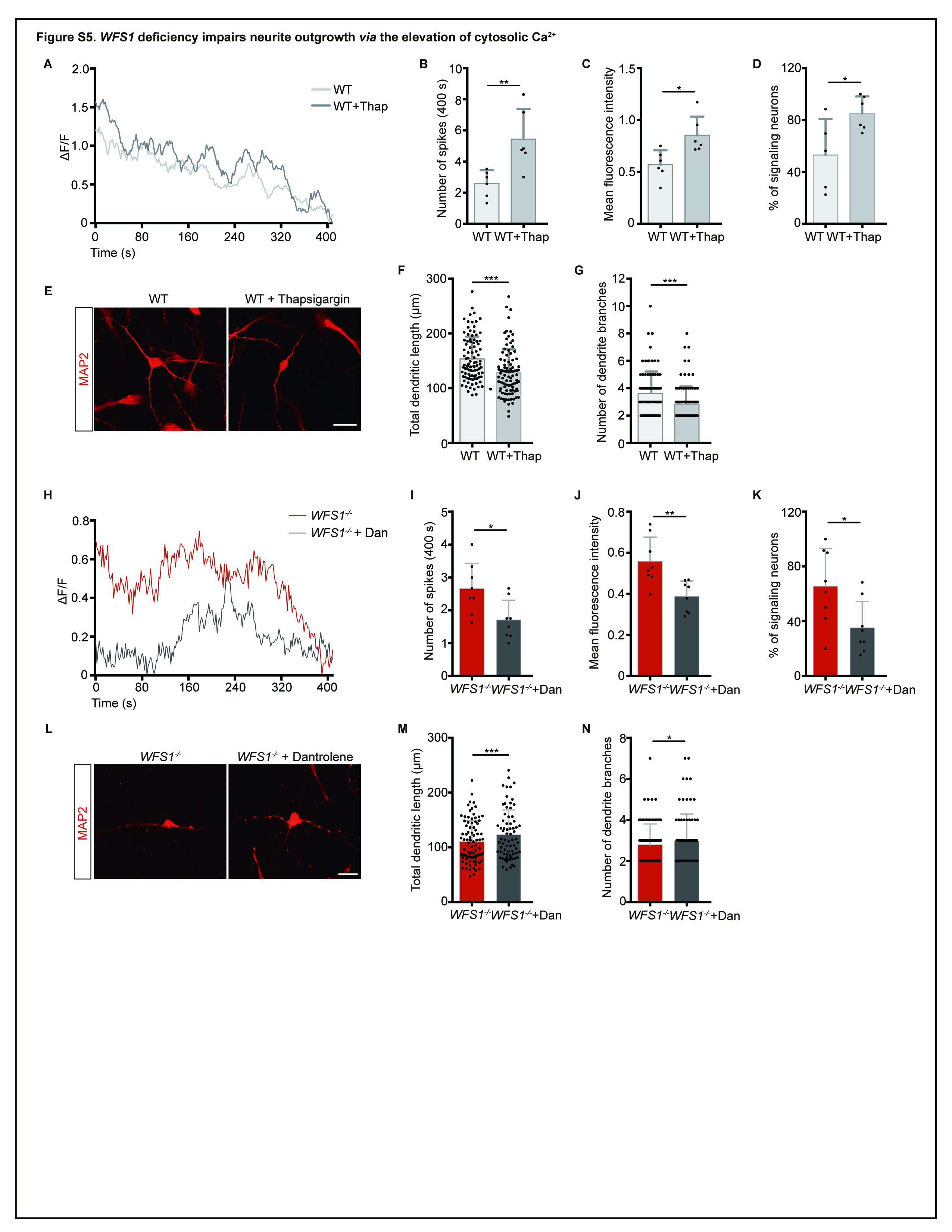

Supplement: Supplementary file 6 — Supplementary figure 5 [file 41380_2023_1987_MOESM6_ESM.tif]

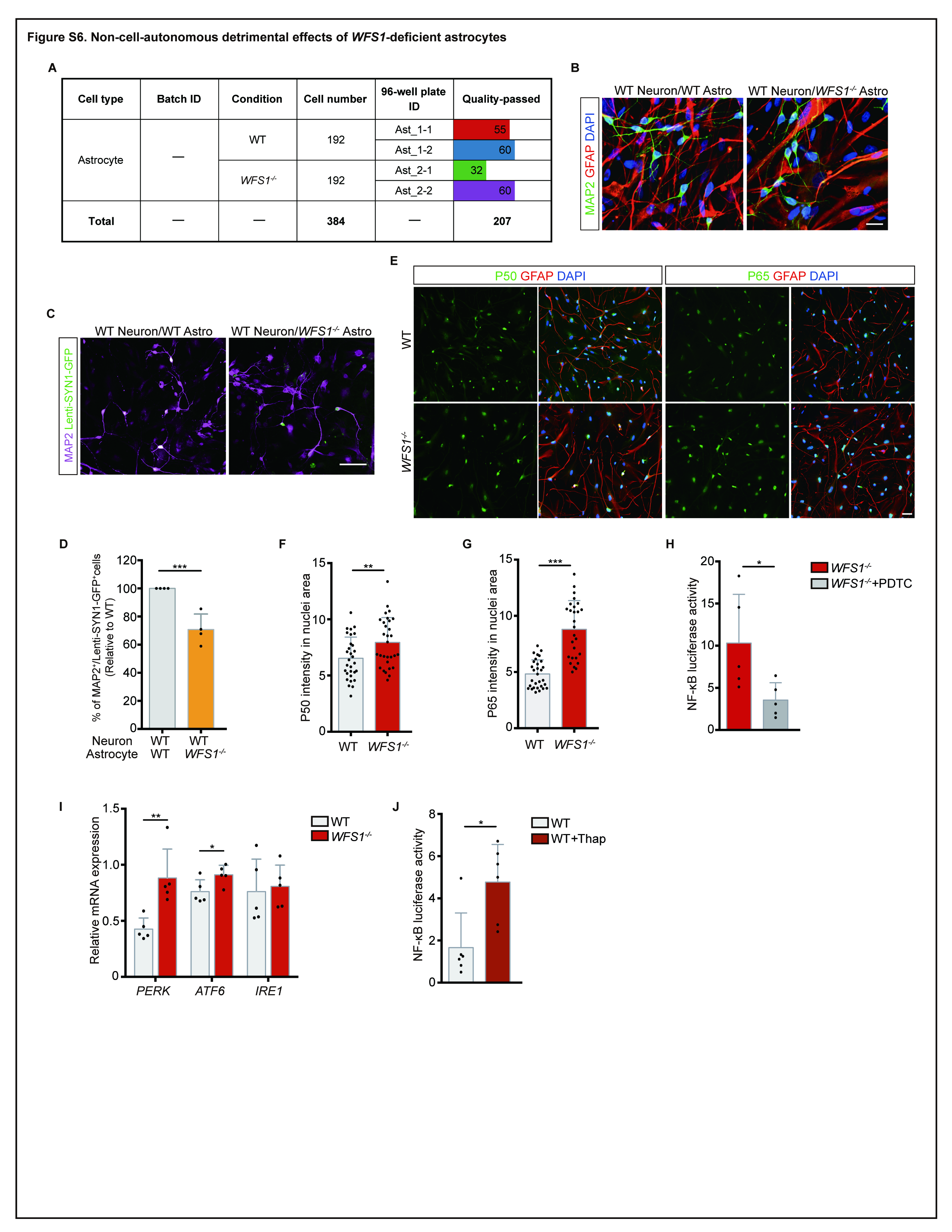

Supplement: Supplementary file 7 — Supplementary figure 6 [file 41380_2023_1987_MOESM7_ESM.tif]

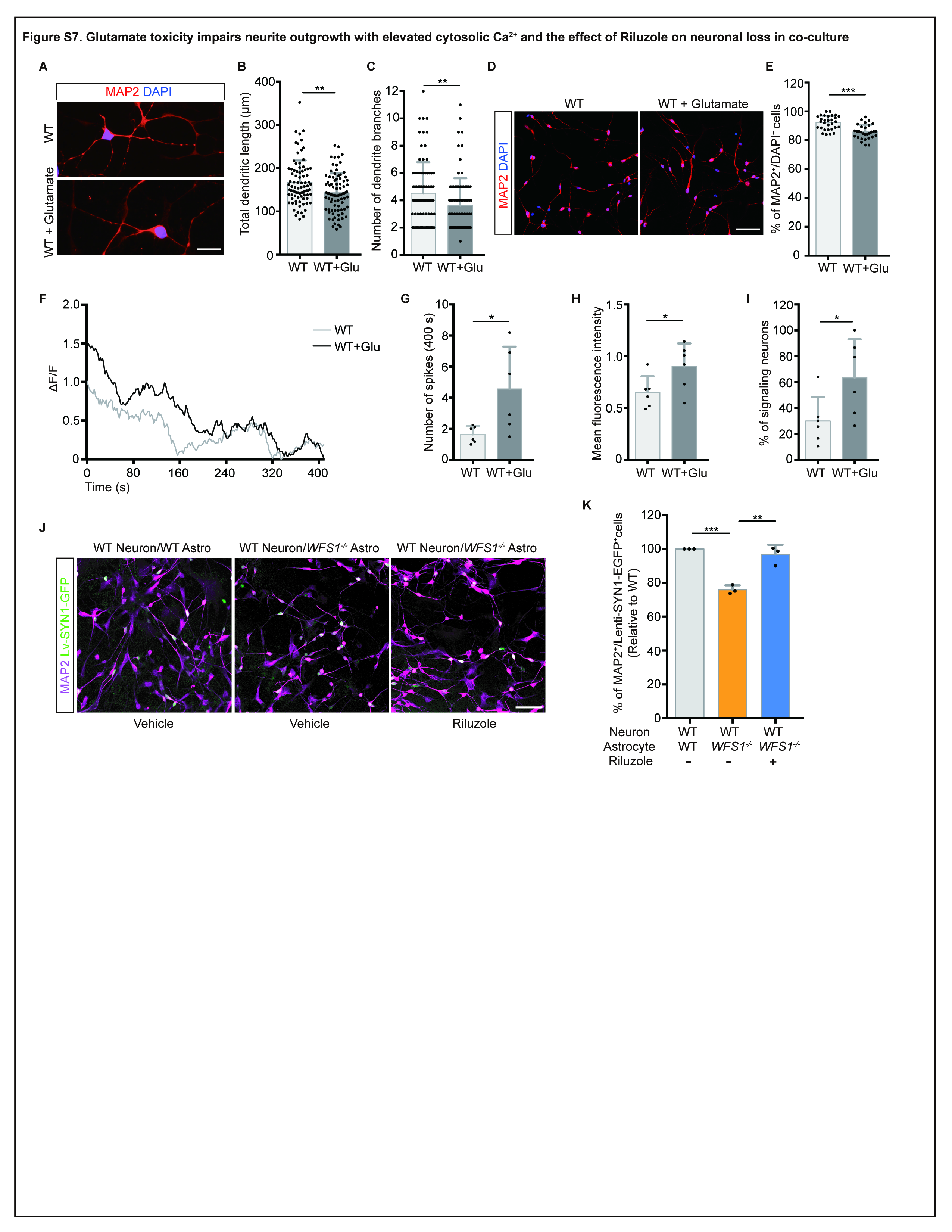

Supplement: Supplementary file 8 — Supplementary figure 7 [file 41380_2023_1987_MOESM8_ESM.tif]
